# STING Agonists and How to Reach Their Full Potential in Cancer Immunotherapy

**DOI:** 10.1002/advs.202500296

**Published:** 2025-03-27

**Authors:** Laura Gehrcken, Christophe Deben, Evelien Smits, Jonas R.M. Van Audenaerde

**Affiliations:** ^1^ Center for Oncological Research (CORE), Integrated Personalized and Precision Oncology Network (IPPON), Faculty of Medicine and Health Sciences University of Antwerp Wilrijk 2610 Belgium

**Keywords:** cancer, immunotherapy, STING agonists, tumor microenvironment, tumor resistance

## Abstract

As cancer continues to rank among the leading causes of death, the demand for novel treatments has never been higher. Immunotherapy shows promise, yet many solid tumors such as pancreatic cancer or glioblastoma remain resistant. In these, the “cold” tumor microenvironment with low immune cell infiltration and inactive anti‐tumoral immune cells leads to increased tumor resistance to these drugs. This resistance has driven the development of several drug candidates, including stimulators of interferon genes (STING) agonists to reprogram the immune system to fight off tumors. Preclinical studies demonstrated that STING agonists can trigger the cancer immunity cycle and increase type I interferon secretion and T cell activation, which subsequently induces tumor regression. Despite promising preclinical data, biological and physical challenges persist in translating the success of STING agonists into clinical trials. Nonetheless, novel combination strategies are emerging, investigating the combination of these agonists with other immunotherapies, presenting encouraging preclinical results. This review will examine these potential combination strategies for STING agonists and assess the benefits and challenges of employing them in cancer immunotherapy.

## Graphical abstract

STING agonists show promise in preclinical studies in boosting an anti‐tumor response using the immune system. However, different limitations remain, and future research is needed to better understand how STING activation impacts the tumor microenvironment, identify effective combination strategies, and determine the best tumor types for STING agonist use to enhance clinical outcomes and overcome immunosuppressive effects.

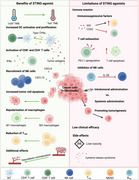

## 1 Introduction

Despite continuous advancements in early diagnostics, drug development, and cancer therapy, cancer remains one of the leading causes of death. Projections suggest that by 2050 the number of cases worldwide will have reached over 35 million, underscoring a significant demand for innovative treatments.^[^ ^1^ ^]^ In this regard, immunotherapies have significantly revolutionized cancer medicine over the past decade, particularly with the advancement of immune checkpoint inhibitors (ICIs), targeting negative regulatory checkpoints.^[^ ^2^ ^]^ However, the efficacy of those inhibitors varies across cancer types and while being successful in several cancer types including melanoma,^[^ ^3^ ^]^ renal cell carcinoma^[^ ^4^ ^]^ lung cancer,^[^ ^3^, ^5^ ^]^ and liver cancer,^[^ ^6^ ^]^ other solid tumors like pancreatic cancer^[^ ^3^, ^7^ ^]^ often do not experience substantial benefits. Besides the breakthrough of ICIs, chimeric antigen receptor (CAR)‐T cell therapies recently also gained significant success, especially with their remarkable efficacy in hematological malignancies. Unfortunately, these results could not yet be extended to solid tumors.^[^ ^8^ ^]^

Reasons for the potential ineffectiveness of immunotherapies are diverse but an important factor is the almost always present immunosuppressive tumor microenvironment (TME), which can be characterized by hypoxia, the presence of anti‐inflammatory immune cells such as regulatory T cells (*T*_regs_), overexpression of immune checkpoints, immune exhaustion with impaired survival and proliferation as well as a poor infiltration of anti‐tumoral immune cells due to a compromised vasculature, causing immune exclusion.^[^ ^9^ ^]^ Additionally, the lack of pre‐existing anti‐cancer immunity presents an additional threshold that must be overcome by advanced immunotherapies.

While ICIs can address the overexpression of immune checkpoints, other challenges require new therapeutics. Consequently, numerous approaches have been investigated to boost the pre‐existing anti‐tumoral immunity, thereby activating the cancer immunity cycle. In this cycle, cancer cell antigens are released first, then taken up by antigen‐presenting cells (APCs), primarily dendritic cells (DCs). Subsequently the APCs present the cancer antigens to T cells in tumor draining lymph nodes, priming and activating them. Activated T cells then migrate and infiltrate the tumor site, recognizing the tumor antigens, leading to the killing of the tumor cells and the release of new antigens.^[^ ^10^ ^]^ Increased intratumoral T cell infiltration is associated with better overall survival in various cancer types, driving the research interest toward stimulating the cancer immunity cycle. For kickstarting the cycle, agonists such as CD40 agonists, Toll‐like‐receptor (TLR) agonists, or cyclic GMP‐AMP synthase (cGAS)‐stimulator of interferon genes (STING) agonists have entered the stage.^[^ ^11^ ^]^ Those agonists can enhance antigen uptake and activation of APCs, thereby leading to an increased T cell response. Specifically, the cGAS/STING pathway as a major regulator of innate immunity holds great promise due to the importance of type I interferons (IFNs). This family of pleiotropic cytokines such as IFNα or IFNβ are increasingly secreted upon activation of this pathway. In contrast, the cytotoxic IFNγ belongs to the type II IFN family. Type I IFNs have been linked with multiple positive effects on anti‐tumoral immune cells such as enhanced cytotoxicity of natural killer (NK) cells, increased intratumoral accumulation, differentiation, and activation of DCs and therefore elevated T cell responses and inducing higher apoptosis of tumor cells.^[^ ^12^ ^]^ In this way, the STING pathway can not only kickstart but also act on later phases of the cancer immunity cycle. During this cycle, DCs can be activated by tumor‐derived DNA, resulting in the activation of STING and due to the produced type I IFNs, DC maturation as well as accumulation increases.^[^ ^13^ ^]^ The activation of STING further mediated the differentiation of naïve T cells into IFNγ‐producing type 1 T helper cells and IL‐9 producing T_H_9 T cells. Both CD4 T cell subsets were needed for the therapeutic effect of cGAMP.^[^ ^14^ ^]^ Moreover, STING activation in DCs enhanced the presentation of tumor‐associated antigens to CD8^+^ T cells on major histocompatibility complex I (MHCI),^[^ ^15^ ^]^ hence amplifying CD8^+^ T cell expansion and trafficking to the tumor site.^[^ ^16^ ^]^ The killing of tumor cells through the CD8^+^ T cells increased the release of additional tumor antigens, further activating DCs, and promoting the initiation of the cancer‐immunity cycle.^[^ ^17^ ^]^

This review focuses on the rationale behind, and the effects mediated by STING agonists in cancer treatment, by exploring how STING agonists have the potential to convert a “cold” tumor site into a pro‐inflammatory “hot” TME, which makes it an interesting partner in immunotherapy. We also discuss limitations that need to be addressed to achieve optimal clinical efficacy.

## 2 STING Agonists: Mode of Action

**Figure 1:**
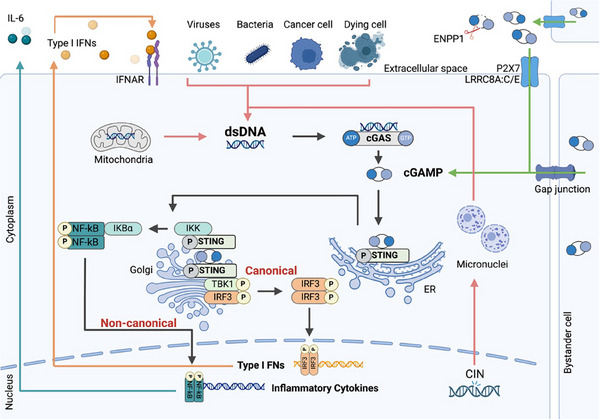
Summary of the cGAS‐STING pathway. Self‐DNA from mitochondria or derived from chromosomal instability (CIN) through micronuclei or foreign double‐stranded DNA (dsDNA) from viruses, bacteria, or cancer can trigger the recognition by cyclic GMP‐AMP synthase (cGAS). The second messenger cyclic guanosine monophosphate‐adenosine monophosphate (cGAMP) binds to the stimulator of interferon genes (STING) on the endoplasmatic reticulum, initiating dimerization. Following TANK‐binding kinase 1 (TBK1) phosphorylation after STING translocation to the Golgi apparatus, TBK1 transphosphorylates interferon regulatory factor 3 (IRF3), which dimerizes and translocates into the nucleus and activates the transcriptional activation of type I interferons (IFNs, orange arrows). Type I IFNs can leave the cell and then bind to the interferon‐α/β receptor (IFNAR) on other immune cells, creating a positive feedback loop. The non‐canonical NF‐κB pathway activates the secretion of inflammatory cytokines such as IL‐6 (turquoise arrows). In addition, cGAMP can enter cells via transporters (P2×7, LRRC8A:C/E) or gap junctions from bystander cells. Red arrows indicate DNA sources activating the cGAS‐STING pathway, while green arrows show cGAMP sources. Ectonucleotide pyrophosphatase/phosphodiesterase 1 (ENPP1) degrades exogenous cGAMP. Figure created with BioRender.

What was initially observed as an evolutionary conserved mechanism against intracellular foreign or self‐DNA, originating from viruses or bacteria, has now also been identified as a critical pathway for anti‐tumoral immunity. STING, broadly expressed in various tissues and cell types, serves as a connection between innate and adaptive immunity. A more detailed overview of the pathway was reviewed elsewhere.^[^ ^18^ ^]^ Shortly in the canonical STING pathway, cGAS functions as an innate immune sensor for double‐stranded DNA (dsDNA), both exogenous and endogenous in an amino acid length‐dependent way^[^ ^19^ ^]^ (**Figure** **1**). Studies have shown, that inactive cGAS remains in the nucleus, tethered to the nucleosome.^[^ ^20^ ^]^ While the exact mechanism is still being investigated, this regulatory system is needed to maintain cGAS inactive, as a continuously active cGAS could promote autoimmunity. Upon detection, cGAS catalyzes both adenosine triphosphate (ATP) and guanosine triphosphate (GTP) into 2′3’ cyclic GMP‐AMP (cGAMP), acting as a second messenger.^[^ ^21^ ^]^ Activation of STING by cGAMP binding to the cyclic dinucleotide (CDN) binding pocket, initiates a cascade leading to the translocation of STING from the endoplasmatic reticulum (ER) to the Golgi apparatus.^[^ ^22^ ^]^ This translocation of STING results in the dimerization and oligomerization as well as the recruitment of downstream effectors, including TANK‐binding kinase 1 (TBK1). TBK1‐mediated autophosphorylation and phosphorylation of STING induces the recruitment of the transcription factor interferon regulatory factor three (IRF3), which then dimerizes, and relocates into the nucleus, binding to IFN‐stimulated response elements (ISRE), mediating the transcriptional activation of interferon‐stimulated genes (ISGs)^[^ ^23^ ^]^ (Figure 1). This activates an autocrine and paracrine production of type I IFNs, mainly IFNα or IFNβ. These can in turn amplify the immune response through positive feedback loops by binding to the interferon‐α/β receptor (IFNAR), expressed on nucleated cells such as B cells, T cells, or cancer associated fibroblasts (CAFs).^[^ ^24^ ^]^ Importantly, the absence of STING or IRF3 leads to a diminished anti‐tumoral immune response and reduced responses to checkpoint blockade.^[^ ^25^ ^]^

In addition to the canonical cGAS‐STING pathway, STING can also activate the non‐canonical (NC) nuclear factor kappa‐light‐chain‐enhancer of activated B cells (NF‐κB) pathway, another key player of the immune system. The activation of the NC NF‐κB pathway is linked to the release of inflammatory cytokines as well as immunosuppressive effects^[^ ^26^ ^]^ or the induction of IL‐6 in various context‐dependent TME scenarios.^[^ ^27^ ^]^ Furthermore, the activation of the NC NF‐κB pathway occurs partly independent of TBK1.^[^ ^28^ ^]^ Yum et al. developed a STING mouse strain with a S365A mutation (S365A), disrupting the IRF3 signaling, demonstrating that STING can function independently of type I IFNs, while requiring TBK1 recruitment for mediating anti‐tumor immunity.^[^ ^29^ ^]^ However, whether NF‐κB interacts directly or indirectly with the STING signalosome remains an open question.

Next to NC NF‐κB pathway activation, exogenous CDNs such as cGAMP produced by bystander cells or cancer cells,^[^ ^30^ ^]^ can either enter the cell via gap‐junctions^[^ ^31^ ^]^ or transporter molecules such as P2×7 or LRRC8A:C/E^[^ ^32^ ^]^ (Figure 1). Exogenous CDNs are highly unstable, and it remains an open research question if a certain threshold of cGAMP needs to be exceeded to activate STING. Furthermore it would be of interest to see which cells in the TME are prone to the uptake and activation of cGAMP.^[^ ^18^ ^]^

In addition to exogenous CDNs, rupture‐prone micronuclei (MN), which are produced especially in tumors with high chromosomal instability (CIN), can be a source of DNA to activate the cGAS‐STING pathway. CIN^high^ tumors are associated with metastasis^[^ ^33^ ^]^ and immune evasion.^[^ ^34^ ^]^ The rupture of the micronuclear envelope in the cytoplasm exposes the genomic dsDNA within, triggering recognition via cGAS and further initiation of the whole cascade^[^ ^35^ ^]^ (Figure 1). Here it is important to note that in CIN^high^ tumors often the NC NF‐kB pathway rather than the STING‐IRF3 pathway is activated, thereby mediating treatment resistance and promoting epithelial‐mesenchymal transition (EMT).^[^ ^33a^ ^]^

The cGAS‐STING pathway can be activated independently of any ligand solely through STING translocation to the Golgi, which can result from STING trafficking gene mutations, supporting the development of autoimmune diseases such as STING‐associated vasculopathy with onset infancy (SAVI) or COPA syndrome.^[^ ^36^ ^]^

## 3 STING Activation and Suppression in the TME

The cGAS/STING pathway is not only important for the defense against pathogens, but also in anti‐cancer immunity, primarily through the secretion of type I IFNs. Cancer cells themselves can activate STING via various mechanisms including the accumulation of DNA induced by chemoradiation, senescence or CIN thereby leading to the production of IFNβ.^[^ ^11^, ^13^ ^]^ Deficiency in STING, demonstrated through knockout studies of *Tmem173* or *Irf3*, correlated with lower T cell infiltration and resistance to ICIs in murine models.^[^ ^25^ ^]^

**Figure 2:**
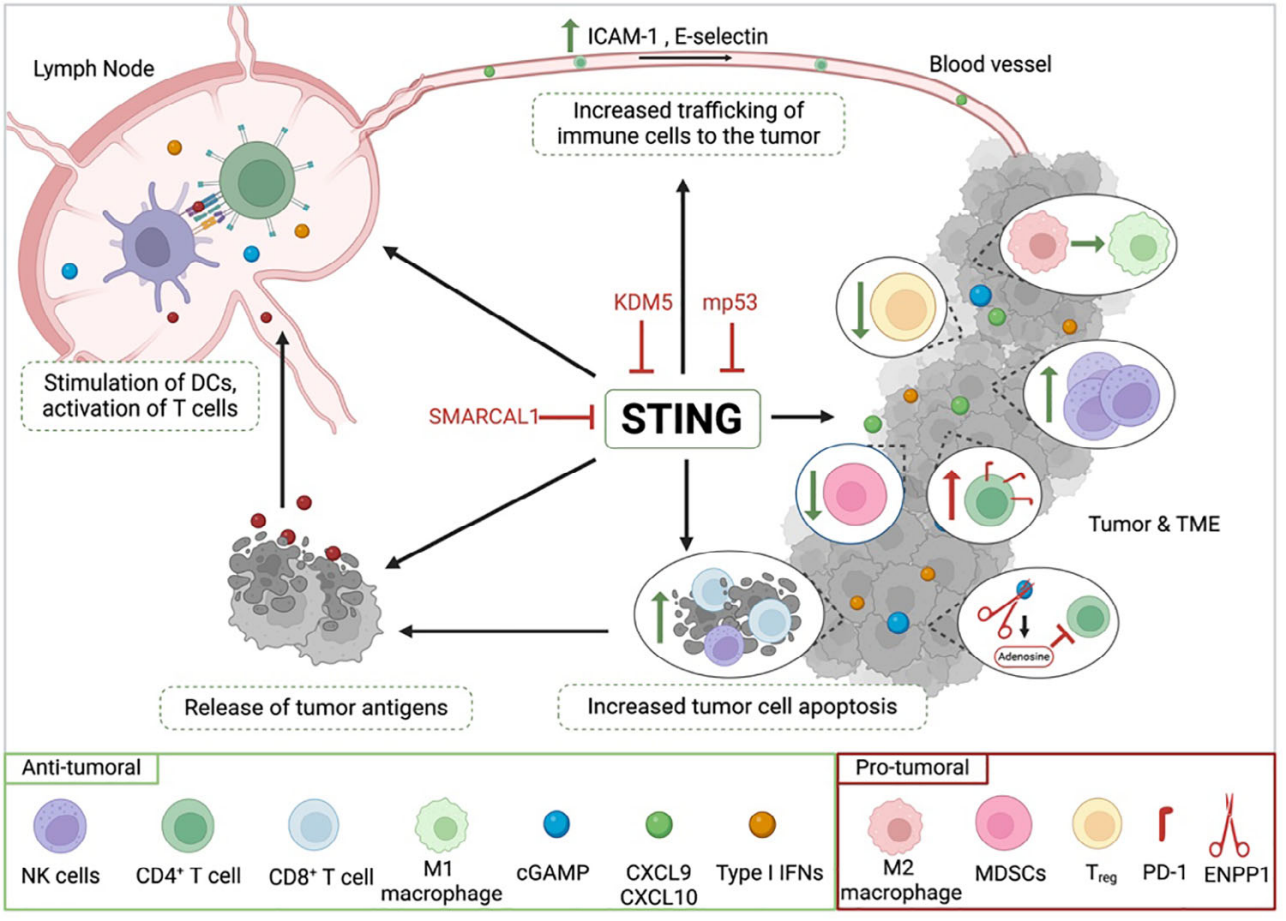
STING activation and suppression within the cancer immunity cycle. Antigens are released in the tumor microenvironment (TME), triggering dendritic cell (DCs) activation in tumor‐draining lymph nodes. These DCs then present tumor antigens to T cells, activating them. STING activation results in ICAM‐1 and E‐selectin upregulation and secretion of chemokines like CXCL9 and CXCL10, enhancing the trafficking of immune cells like T cells or natural killer (NK) cells into the TME. Inside the tumor, STING activation boosts NK cells, and increases CD4^+^ and CD8^+^ T cells while decreasing regulatory T cells (*T*_regs_) and myeloid‐derived suppressor cells (MDSCs). The rise in cytotoxic CD8^+^ T cells promotes tumor cell death, which in turn increases the release of tumor antigens, perpetuating the cancer immunity cycle. However, STING activation in the TME can lead to PD‐1 upregulation on T cells, while ENPP1 can degrade cGAMP into adenosine, inhibiting T cell function. Tumor cells often modify STING via methylations through KDM5 or inhibit STING activation via SMARCAL or mutated p53, blocking the STING‐TBK1‐IRF3 complex. Figure created with BioRender.

STING‐driven immunity is also mediated by NK cells, where tumor derived cGAMP enhanced NK cell activity upon STING pathway stimulation^[^ ^37^ ^]^ (**Figure** **2**). STING agonist treatment increased NK cell migration in malignant pleural mesothelioma, correlating with increased tumor cell death.^[^ ^38^ ^]^ Furthermore, a depletion of STING reduced NK cell recruitment and activation in NK cell‐sensitive tumors, such as B16D8 melanoma.^[^ ^39^ ^]^ Additionally, STING activation promoted the secretion of the C‐X‐C motif chemokine receptor 3 (CXCR3)‐binding chemokine C‐X‐C motif chemokine ligand 9 (CXCL9) and CXCL10 as well as C‐C motif ligand 5 (CCL5) by tumor cells and CAFs, promoting NK and T cell recruitment (Figure 2).^[^ ^16^, ^38^ ^]^ Interestingly, Wolf et al. showed that a combination of intratumoral CDN together with the IL‐2 superkine H9‐MSA induced strong systemic activation of NK cells.^[^ ^40^ ^]^ They demonstrated that CD8^+^ T cells, but not NK cells, were required for MHC‐I^+^ tumor elimination. Conversely, in MHC‐I‐deficient tumors, NK cells, more than CD8^+^ T cells were required for effective tumor elimination.^[^ ^40^ ^]^ Additionally, intratumoral cGAMP treatment of B16‐F10 tumors with high STING protein expression was associated with NK cell‐mediated tumor growth inhibition, whereas in 4T1 breast cancers with low STING expression, cGAMP monotherapy was insufficient to stop tumor growth.^[^ ^41^ ^]^ These data highlight the indirect effect of STING agonists on NK cell activation in the presence of STING‐expressing tumor cells.

Besides mediating the anti‐tumoral immune response, cGAS‐STING pathway activation demonstrated modulatory effects on the TME. It has been shown to repolarize immunosuppressive M2 into anti‐tumoral M1 macrophages^[^ ^42^ ^]^ as well as reducing the number of *T*_regs_.^[^ ^16b^ ^]^ In addition, STING deficiency correlated with increased numbers of CD11b^+^Gr‐1^+^ immature myeloid‐derived suppressor cells (MDSCs) and CD25^+^FoxP3^+^ *T*_regs_^[^ ^16b^ ^]^ (Figure 2).

Another crucial factor to increase the anti‐tumoral immune response is to enhance immune cell extravasation into immune‐deserted tumor sites by upregulating factors like E‐selectin, intercellular adhesion molecul 1 (ICAM‐1), or vascular cell adhesion molecule 1 (VCAM‐1) due to the STING pathway activation.^[^ ^43^ ^]^ Additionally, a combination of cGAMP and anti‐vascular agents could improve the response of tumors with low STING‐protein expression to therapy mediated via NK cells.^[^ ^41^, ^44^ ^]^ Consequently, the ability to activate the immune system and trigger an anti‐tumoral immune response thereby converting an immune‐deserted environment into a “hotter”, more immunogenic TME, highlights the STING pathway activation as a promising target in cancer therapy. Therefore, efforts have been focused on evaluating the potential of STING agonists as an immunotherapy in cancer as reviewed later.

However, since activating the cGAS‐STING pathway through damaged DNA is unfavorable for cancer cells, many tumors have developed diverse strategies to evade detection and suppress the immune response with the primary objective of stabilizing the genome and prevent MN formation^[^ ^33a^ ^]^ (Figure 2). To limit MN generation, SMARCAL1^[^ ^45^ ^]^ suppresses the cGAS‐STING pathway by reducing endogenous DNA damage while for example, MUS81 helps maintain genomic integrity.^[^ ^46^ ^]^ Mutant p53 degraded TREX1, a cytosolic nuclease responsible for clearing DNA and preventing cGAS activation. Therefore, DNA accumulated and activated STING.^[^ ^47^ ^]^ However, mutant p53 interacted with TBK1 and destabilized the STING‐TBK1‐IRF3 complex, promoting immune evasion.^[^ ^48^ ^]^ Moreover, different histone modifications such as epigenetic suppression of STING via the histone H3K4 lysine demethylase KDM5 in breast cancer cells^[^ ^49^ ^]^ or a STING promoter hypermethylation silencing STING expression^[^ ^50^ ^]^ are utilized by the cancer cells to repress STING. This has been comprehensively reviewed elsewhere.^[^ ^18^ ^]^ Nevertheless, not all tumors downregulate STING, as seen in unstable esophageal cancer. Despite displaying high CIN, the cGAS‐STING pathway is largely maintained. Instead of the type I IFN response, chronic and transient STING activation promotes the expression of pro‐inflammatory cytokines, indicating a more inflammatory TME.^[^ ^51^ ^]^

Since exogenous cGAMP serves as an activator for the pathway, it is important to note that it can be rapidly degraded by ecto‐nucleotide pyrophosphatase/ phosphodiesterase 1 (ENPP1)^[^ ^52^ ^]^ (Figure 1). A recent study showed that ENPP1 drives breast cancer growth by reducing extracellular cGAMP.^[^ ^52a^ ^]^ The breakdown of extracellular cGAMP enhanced the concentration of adenosine in the TME, reduced immune cell infiltration, and increased metastasis in CIN tumors^[^ ^53^ ^]^ (Figure 2).

The mechanisms of cGAS‐STING pathway suppression by tumor cells are diverse and might be either direct or indirect. Nevertheless, those modifications do not lead to a complete absence of STING as mutations in the *CGAS* or *TMEM173*, the STING encoding gene, are rare (0.5–0.6%) in CIN tumors.^[^ ^54^ ^]^ Therefore, inhibitors of DNA methylations or ENPP1 might be beneficial co‐stars to improve the STING‐related anti‐tumor response.

## 4 STING Agonists and Their Positive Effects on the TME

**Figure 3:**
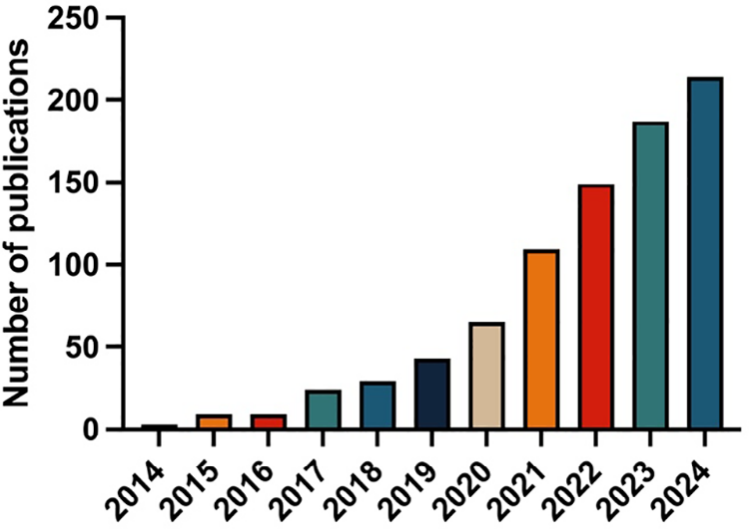
Number of publications in Pubmed when searching for "STING agonist" and "cancer" over the last nine years. Checked in February 2025. Both review and research articles are included.

As seen above, STING activation in the TME results in the beneficial activation of immune cells, which fueled the interest in STING agonists over the last decade (**Figure** **3**). Consequently, it is not surprising that multiple STING agonists have been developed and investigated in preclinical research and clinical trials.

Currently available STING agonists can be categorized into two groups: CDNs and synthetic STING agonists. Both CDNs and synthetic STING agonists mimic the natural interaction between the STING ligand cGAMP and STING.

Natural CDNs such as 3′,5′‐diguanylic acid (c‐di‐GMP) or monophosphate (2′3‐cGAMP or 3′3’‐cGAMP), showed anti‐tumor efficacy,^[^ ^11^, ^55^ ^]^ however their intrinsic physiochemical characteristics like electronegativity and hydrophilicity as well as their susceptibility to enzymatic degradation by the phosphodiesterase ENPP1 limited their bioavailability within tumor tissues and narrowed the therapeutic window.^[^ ^56^ ^]^ Hence, synthetic STING agonists, such as ADU‐S100 or BMS‐986301 are engineered for better stability and efficacy, thereby circumventing the disadvantages of natural CDNs.

5,6‐dimethylxanthenone‐4‐acetic acid (DMXAA) was one of the first synthetic STING agonists, initially identified as a potent tumor vascular disruptor,^[^ ^57^ ^]^ which entered clinical trials due to encouraging in vivo data.^[^ ^58^ ^]^ In preclinical models, DMXAA demonstrated the capability to reduce tumor burden and increase the intratumoral secretion of inflammatory cytokines in an in vivo pancreatic cancer model.^[^ ^58^ ^]^ However, despite these promising preclinical results, clinical trials failed, as DMXAA could not engage with human STING, ultimately reducing its clinical efficacy.^[^ ^59^ ^]^ Notably, a recent publication shed light on this issue, demonstrating that DMXAA is a partial STING agonist, interfering with agonistic STING activation by suppressing the STING‐induced anti‐tumor effect.^[^ ^60^ ^]^

Currently, many of the newly developed synthetic STING agonists are able to bind all or most common isoforms of human STING resulting in increased production of type I IFNs leading to enhanced T cell activation,^[^ ^61^ ^]^ followed by tumor regression^[^ ^62^ ^]^ in various mouse models such as colorectal cancer^[^ ^62^, ^63^ ^]^ or acute myeloid leukemia (AML)^[^ ^62a^ ^]^ (as summarized in **Table** **1**). A more detailed overview of different STING agonists in preclinical studies was reviewed elsewhere.^[^ ^11c^ ^]^

**Table 1:** Overview of different STING agonists and their characteristics from preclinical and if applicable from clinical studies. IT – intratumoral; IM – intramuscular; IV – intravenous; SC – subcutaneous; IP – intraperitoneal; PO – oral; AML – acute myeloid leukaemia.

Agonist	Route of administration	Findings	Reactivity	Reference
DMXAA	IT	Potent anti‐tumor response in mice, ineffective in humans, increased number of CD8^+^ T cells	Mouse	[58, 68]
ADU‐S100 (MIW815)	IT	Increased clonal T cell expansion, increased IFNγ and CXCL10 in advanced and metastatic tumors, limited clinical activity but well tolerated	Human/ mouse	[11, 61]
SHR1032	IT	Higher activity than ADU‐S100 on human AML cells, strong anti‐tumor effects, increased production of TNFα, IFNβ and IL‐6 in mouse MC38 cancer	Human	[62a]
SB11285	IT	Induced potent and durable anti‐tumor responses in A20 lymphoma and colorectal cancer mouse model	Human/ mouse	[62b]
BMS‐986301	IM/ IT	Induction of type I IFNs, low T cell toxicity and in combination with an anti‐PD1 antibody up to 80% remission in CT26 and MC38 mouse model, IM potentially superior to IT administration	Human/ mouse	[69]
E7766	IT	Enhanced potency, increased survival, and long‐lasting immune response in a SC CT26 mouse model, potential for pan‐genotypic activity	Human/ mouse	[62c]
TAK‐676	IV	Enhanced DC recruitment and maturation, dose‐dependent DC, T & NK cell increase, no tumor regrowth in B16‐F10 tumor‐bearing mice	Human/ mouse	[62d]
GSK3745417	Not reported	Cell growth inhibitory effect on human AML cell lines and patient samples with type I IFN production in all cell lines	Not reported	[70]
MK‐1454	IT	Potent in cell‐based assays, similar binding characteristics as 2′3’‐cGAMP, reduced tumor volume in MC38 and B16‐F10 mouse tumor model	Human/ mouse	[71]
SNX281	IV	Induced tumor regression in CT26 mouse model, well tolerated, expected higher cellular potency in an acidified environment	Human/ mouse	[72]
diABZI	IV	Increased survival and decreased tumor volume in CT26 mouse model, increase of CD8^+^ T cells, increased tumor antigen presentation and levels of IL‐6 and TNF	Human/ mouse	[61, 64]
MSA‐2	PO	Effective in low pH regions, tested in MC38 and CT26 mouse models, in combination with anti‐PD‐1 in a mouse model with poor anti‐PD‐1 response increased survival and CD8^+^ T cells	Human/ mouse	[63]
SR‐717	IP	Decreased tumor burden, increased amount of CD8^+^ T cells and NK cells, upregulation of PD‐L1 and IDO in B16‐F10 mouse model	Human/ mouse	[65]

A notable limitation of multiple STING agonists is their requirement for intratumoral (IT) administration. While this approach works for tumors that are easily accessible, it proves to be inadequate for most solid tumors. Therefore, investigations have focused on systemic STING agonists, which carry a risk of causing cytokine release syndrome and acute inflammation if their activity is not restricted to the tumor site as well as the potential for overactivation of immune cells, which might induce adverse effects as discussed later. Nevertheless, Compound 3 (diABZI), for instance, induced potent T cell responses and improved overall survival in a subcutaneous CT26 colorectal cancer mouse model after intravenous (IV) injection.^[^ ^61b^ ^]^ Additionally, diABZI showed increased tumor cell apoptosis in vitro and in vivo together with an enhanced expression of tumor antigens.^[^ ^64^ ^]^ Similarly, MSA‐2, another systemic STING agonist, holds promise for inducing tumor regression through oral administration. Functioning as a prodrug, MSA‐2 remains inactive in a physiologic pH of 7.4 but becomes active in the TME, which is characterized by a lower acidic pH (ranging from 6.0–6.9). While this drug has not yet entered clinical trials this strategy might be an appealing way to minimize the associated risks of a systemic STING agonist treatment.^[^ ^63^ ^]^ Furthermore, SR‐717 demonstrated anti‐tumor activity in a melanoma mouse model after intraperitoneal (IP) delivery leading to increased CD8^+^ T cell and NK cell numbers in tumor‐draining lymph nodes as well as the spleen.^[^ ^65^ ^]^ The expression of CD107a as a marker for CD8^+^ T cell degranulation and NK cell activity was upregulated in both cell types within the tumor and spleen. However, the cytotoxic granzyme B was only upregulated in T cells in both tissues, with no increase observed in NK cells. These preclinical results highlight the potential of systemic STING agonists with careful consideration of side effects.

Next to augmenting the anti‐tumoral immune response, STING agonist treatment also blocked abnormal vessel formation, upregulated endothelial‐leucocyte adhesion molecules, and restored peritoneal anti‐tumor activity in colon cancer,^[^ ^42^, ^66^ ^]^ an effect that was not observed in STING knockout mice or mice where IFNAR was blocked, indicating that vasculature normalization is mediated by the STING pathway. In highly vascularized tumors such as 4T1 (colorectal cancer) combining a STING agonist with a vascular disrupting agent (CA4P) strengthened the anti‐tumor response via cGAS‐STING activation.^[^ ^41^ ^]^ Furthermore, in a murine model of melanoma and colon cancer, cGAMP treatment identified endothelial cells rather than DCs as the primary source of type I IFNs,^[^ ^16^, ^67^ ^]^ highlighting a role for the tumor vasculature in the initiation of the CD8^+^ T cell response. Additionally, low‐dose IT ADU‐S100 treatment increased the production of anti‐angiogenic (e.g., CXCL10 or vascular endothelial growth inhibitor) and tertiary lymphoid structure‐inducing factors (e.g., CCL19 and CCL21) in a B16‐F10 melanoma mouse model.^[^ ^67^ ^]^ Treatment efficacy could be even more enhanced upon the combination of a STING agonist with an anti‐vascular endothelial growth factor receptor 2 (VEGFR2) antibody and either an anti‐PD‐1 or anti‐CTLA‐4 antibody compared to mono‐ or dual combination therapy in a colon cancer mouse model.^[^ ^66^ ^]^

These preclinical studies highlight the advantageous potential of STING agonists in cancer therapy. However, variations in mouse models, the types of STING agonists used and their route of administration, and/or which specific immune cells are investigated if any, pose challenges in comparing results across studies. With the growing number of agonists being developed, a standardized way to analyze efficacy and overall impact on the immune system is needed. This would allow researchers to better understand the underlying mechanisms of action and potential toxicities associated with these compounds.

## 5 STING Agonists in Clinical Trials

### 5.1 STING Agonists in Clinical Trials as a Monotherapy

The journey of STING agonists into clinical trials started with DMXAA, representing a milestone. Despite being the first STING agonist ever investigated in a clinical trial for cancer treatment, DMXAA failed to induce a clinical response due to its inability to bind human STING.^[^ ^68^ ^]^ Ever since a couple of clinical trials with STING agonists in a monotherapy setting have been started (**Table** **2**). In a phase I dose‐escalation trial involving 47 patients with advanced/metastatic solid tumors or lymphomas, IT administration of ADU‐S100 was well tolerated and demonstrated limited single‐agent activity. Nevertheless, systemic immune activation was detectable due to a dose‐dependent production of IFNγ and CXCL10 as well as increased clonal T cell expansion.^[^ ^61a^ ^]^ Due to promising preclinical in vivo data,^[^ ^62c^ ^]^ E7766 entered a clinical phase I trial (NCT04109092) but the trial was withdrawn without the enrolment of any patients due to the company's decision, as was their other trial with the same compound (NCT04144140). Currently, two active clinical trials are underway to investigate the effect of SB11285 in advanced solid tumors (NCT04096638) and GSK3745417 for relapsed or refractory myeloid malignancies (NCT05424380). However, despite the potential of STING agonists, their application as monotherapy faces limitations, reflected in the relatively low number of ongoing clinical trials.

**Table 2:** Completed and ongoing clinical trials of STING agonists as a monotherapy for cancer treatment sorted regarding their completion or termination date. Source: Clinicaltrial.gov, October 2024, DLT – delayed immune‐related; AE – adverse events; IVES – intravesical (into the bladder); IT intratumoral; IM – intramuscular; NR – not reported.

STING agonist	Indication	Phase	Route of administration	NCT number	Status	Safety	Results
MK‐1454 (Ulevostinag)	Advanced/ metastatic solid tumors or lymphomas	I	IT	NCT03010176	Completed	well tolerated, AE in 83%, 9% ≥ 3 AEs	No complete or partial responses^[^ ^73^ ^]^
E7766 (INSTAL‐101)	Solid tumors or lymphomas	I	IT	NCT04144140	Terminated	NR	No clinical activity
ADU‐S100 (MIW815)	Advanced/ metastatic solid tumors or lymphomas	I	IT	NCT02675439	Completed	well tolerated, grade 3/4 AEs in 40%, no MTD	No anti‐tumor activity^[^ ^61a^ ^]^
E7766 (INSTAL‐102)	Non‐muscle invasive bladder cancer	I/Ib	IVES	NCT04109092	Withdrawn	NR	NR
CDK‐002 (exo‐STING)	Advanced/metastatic recurrent injectable solid tumors	I/II	IT	NCT04592484	Completed	NR	NR
MK‐2118	Advanced/ metastatic solid tumors or lymphomas	I	IT	NCT03249792	Completed	NR	NR
IMSA101	Refractory malignancies	I/II	IT	NCT04020185	Completed	TEAE in 63,3%, DLT of arthropathy in 1 patient	Notable efficacy signals^[^ ^74^ ^]^
BMS‐986301	Advanced solid cancers	I	IM	NCT04020185	Completed	NR	NR
SB11285	Advanced solid tumors	I	IV	NCT04096638	Recruiting		
GSK3745417	Relapsed or refractory myeloid malignancies	I	IV	NCT05424380	Active, not recruiting		

### 5.2 Combination of STING Agonists with Other Immunotherapies

#### 5.2.1 In Combination with Immune Checkpoint Inhibitors

Numerous ongoing clinical combination strategies involving STING agonists include ICIs (**Table** **3**). The main targeted immune checkpoint pathways include B7.1 (CD80)/CTLA‐4 or B7.2 (CD86)/CTLA‐4 and PD‐L1/PD‐1. These checkpoint axes play a pivotal role in preventing over‐ or chronic activation of T cells. While CTLA‐4 expressed on APCs competes with CD28 for binding with B7.1 or B7.2 on T cells, PD‐L1 is predominately upregulated on tumor cells, inhibiting the activation of T cells as such by interacting with PD‐1 expressed on the latter.^[^ ^75^ ^]^ ICIs targeting PD‐1 include pembrolizumab, nivolumab, spartalizumab, ezabenlimab, or dostarlimab, while atezolizumab, avelumab, or durvalumab target PD‐L1, whereas ipilimumab targets CTLA‐4. They disrupt the negative feedback loop and prevent T cell suppression. Since pre‐existing cytotoxic T cell activation and infiltration into the TME is important for ICIs to work properly, the potential synergism between STING agonists and ICIs lies firstly in their capacity to increase type I IFNs and thereby T cell infiltration, and secondly, in blocking checkpoints on immune cells potentially upregulated by the tumor and STING agonists. Preclinical investigations demonstrated promising results using different STING agonists and ICIs in various mouse models. For instance, the oral STING agonist MSA‐2 combined with an anti‐PD‐1 antibody (muDX400) exhibited synergistic inhibition of tumor growth and prolonged survival in different mouse models (colorectal MC38 and CT26, melanoma B16‐F10 and lung LL‐2 cancer).^[^ ^63^ ^]^ Similarly, a study investigating the combination of an anti‐TGF‐β/PD‐L1 bispecific antibody (YM101) with MSA‐2 demonstrated a synergistically enhanced anti‐tumoral response in multiple murine tumor models (colorectal CT26, liver H22, melanoma B16, and breast EMT‐6 cancer) alongside promoting DC maturation, macrophage reprogramming (M2 to M1) and cytokine/ chemokine stimulation.^[^ ^76^ ^]^ Furthermore, the combination of an ADU‐S100 analogue and an anti‐PD‐1 antibody enhanced the response to carboplatin chemotherapy in a serous high‐grade ovarian cancer mouse model.^[^ ^77^ ^]^ Additionally, IT administration of ADU‐S100 in combination with spartalizumab demonstrated partial responses in patients with PD‐1‐naïve triple‐negative breast cancer and PD‐1‐relapsed melanoma, while the treatment was well tolerated (NCT03172936). Nonetheless, due to a sponsor's decision, the clinical trial was prematurely terminated. These preclinical results indicate potential for the treatment combination; however, clinical trial outcomes have been unsatisfying so far.^[^ ^78^ ^]^ The lack of results in clinical trials for this combination treatment may be from variations in PD‐1 expression induced through the STING agonist. These factors should be taken into account before applying treatment to ensure the best possible outcomes.^[^ ^41^ ^]^

**Table 3:** Completed clinical trials at the top and ongoing clinical trials below using STING agonists in combination with ICIs for different tumor types. Route of administration refers to the STING agonists. Source: clinicaltrial.gov, October 2024; NSCLC – non‐small cell lung cancer; TNBC – triple‐negative breast cancer; SCCHN – squamous cell carcinoma of the head and neck; RCC – renal cell carcinoma; IT – intratumoral; IV – intravenous; IM – intramuscular, SD – stable disease.

STING agonist	Indication	Phase	Route of administration	NCT number	Status	Results
ADU‐S100 (MIW815) + Ipilimumab	Advanced/ metastatic solid tumors, lymphomas	I	IT	NCT02675439	Terminated	No substantial tumor activity
ADU‐S100 (MIW815) + Spartalizumab	Advanced/ metastatic solid tumors, lymphomas	Ib	IT	NCT03172936	Terminated	Partial responses^[^ ^79^ ^]^
ADU‐S100 (MIW815) + Pembrolizumab	Head and Neck cancer (PDL1^+^ or metastatic)	II	IT	NCT03937141	Completed	No substantial tumor activity
SYNB1891 + Atezolizumab	Advanced/ metastatic solid tumors, lymphomas	I	IT	NCT04167137	Terminated	SD in 4/24 patients^[^ ^80^ ^]^
MK‐1454 (Ulveostinag) + Pembrolizumab	Advanced/ metastatic solid tumors, lymphomas	I	IT	NCT03010176	Completed	Combination good efficacy, safe
MK‐1454 (Ulveostinag) + Pembrolizumab	Head and neck cancer	II	IT	NCT04220866	Completed	Not reported
MK‐2118 + Pembrolizumab	Advanced/ metastatic solid tumors, lymphomas	I	IT	NCT03249792	Completed	Not reported
IMSA101 + ICI	Refractory malignancies	I/II	IT	NCT04020185	Completed	Notable efficacy signals^[^ ^74^ ^]^
SNX281 ± Pembrolizumab	Advanced solid tumors and lymphoma	I	IV	NCT04609579	Terminated	Safety concerns
BI 1 387 446 + Ezabenlimab	Advanced/ metastatic solid tumors	I	IT	NCT04147234	Completed	Not reported
BMS‐986301 + Nivolumab + Ipilimumab	Advanced solid tumors	I	IV, IM, IT	NCT03956680	Completed	Not reported
TAK‐676 + Pembrolizumab	NSCLC, TNBC, SCCHN	I	IV	NCT04879849	Completed	Not reported
GSK3745417 ± Dostarlimab	Advanced solid tumors	I	NR	NCT03843359	Active, not recruiting	
SB11285 ± Atezolizumab	Advanced solid tumors	Ia/Ib	IV	NCT04096638	Recruiting	
Dazostinag (TAK‐676) + Pembrolizumab	Advanced/ metastatic solid tumors	I/II	IV	NCT04420884	Recruiting	
TAK500 + Pembrolizumab	Locally advanced or metastatic solid tumors	I	IV	NCT05070247	Recruiting	
IMSA101 ± PULSAR ICI	NSCLC and RCC	II	IT	NCT05846646	Recruiting	
	Oligoprogressive solid tumors	II	IT	NCT05846659	Recruiting	

#### 5.2.2 In Combination with Toll‐like Receptor Agonists

Apart from ICIs, STING agonists have also been investigated in combination with TLR agonists. TLRs are key molecules of the innate immune system, detecting pathogen‐associated patterns or danger‐associated patterns secreted by infected or dying cells. Recognition of these patterns by TLRs triggers downstream pathways, ultimately leading to the production of pro‐inflammatory cytokines, the maturation, and the activation of APCs.^[^ ^11b^ ^]^ Preclinical data demonstrated the anti‐tumor activity of TLR agonists in multiple cancer types^[^ ^11b^ ^]^ while two TLR agonists, Imiquimod (TLR7 agonist) and Bacillus Calmette‐Guérin (BCG) (TLR2/4 agonist) have been approved by the Food and Drug Administration (FDA). The administration of these agonists resulted in increased expression of MHC class II complexes on macrophages and DCs, thereby promoting the secretion of pro‐inflammatory cytokines such as tumor necrosis factor alpha (TNFα) or IFNγ.^[^ ^81^ ^]^ Therefore, combining TLR and STING agonists simultaneously heightened both type I IFNs as well as other pro‐inflammatory cytokines and additionally increased the maturation and activation of APCs, turning a “cold” TME into a “hot” one.^[^ ^11b^ ^]^

In preclinical investigations, the TLR9 agonist K3 CpG and the STING agonist 3′3’cGAMP demonstrated a synergistic effect in inducing IFNγ and enhanced CD4^+^, CD8^+^ T cell as well as NK cell responses, ultimately suppressing tumor growth in lymphoma and melanoma in vivo.^[^ ^82^ ^]^ Similarly, the combination of ADU‐S100 with CpG ODN1826 (TLR9 agonist) showed decreased tumor volume and an improved survival rate in a SC CT26 model compared to the PBS control group. Furthermore, a combination of the STING and the TLR agonist induced a downregulation of alpha smooth muscle actin (αSMA) and vimentin (VIM) which are important markers for CAFs, suggesting that the combination strategy has the potential to suppress tumor‐promoting effects of CAFs in a mouse model of colon cancer.^[^ ^83^ ^]^ Of note, the underlying mechanisms for this effect will require further investigation. Additionally, two cycles of IV administration of a STING agonist in a lipid nanoparticle together with CpG ODN increased the number of CD11b^high^CD27^low^ memory NK cells in a murine melanoma model, thereby highlighting the potential effect of STING agonists on memory NK cells and the possible importance of administering multiple doses.^[^ ^84^ ^]^ Furthermore, combining a TLR7/8 agonist (‘522′) and DMXAA decreased tumor volume and M2 macrophages while NK cells were increased.^[^ ^85^ ^]^ Notably, pre‐activation of the STING pathway suppressed the TLR9‐mediated IFN production from plasmacytoid DCs. This finding underscores the importance of administering both agonists at the same time to prevent antagonisms. Overall, the synergistic mechanism underlying the combination of STING and TLR agonists holds promise to enhance immune activation and overcome tumor‐mediated resistances, thereby improving anti‐tumor responses. Despite the promising preclinical data, no combination of STING and TLR agonists has entered clinical trials yet.

#### 5.2.3 In Combination with Adoptive Cell Therapies

Next to ICIs and TLR agonists, another potential combination involves leveraging the benefits of adoptive cell therapies. These therapies include CAR‐transduced cell therapy, T cell receptor (TCR)‐T cell therapy, or tumor‐infiltrating lymphocyte (TIL) therapy. To date, the majority of research has focused on combining STING agonists with CAR‐transduced cells. Combining the specific targeting of antigens via CAR‐T cells or CAR‐NK cells along with a STING agonist creates an effective therapeutic strategy, as this approach could amplify the trafficking of CAR‐transduced cells to the tumor site and further increase their anti‐tumoral activity. Since target antigen loss is a significant challenge associated with CAR cell therapy, the use of STING agonists in combination could prevent this tumor escape mechanism.^[^ ^86^ ^]^ A preclinical study using different CAR‐T cells (anti‐CD19‐, anti‐gp75, and anti‐PSMA CAR‐T cells) alongside IT administration of 2′3’ cGAMP demonstrated increased CAR‐T cell killing, resulting in improved overall survival and reduced tumor volume in a melanoma mouse model. Furthermore, the combination treatment counteracted the loss of antigen expression by epitope spreading.^[^ ^86^ ^]^ Combining DMXAA or 2′3’ cGAMP with LH28z‐CAR‐Th/Tc17 cells led to an increase of CAR‐T cells at the tumor site, even when the agonist was injected distally rather than intratumorally in an orthotopic breast cancer model. Although the STING agonist alone was not able to prevent exhaustion of the T cells, a combination with an ICI proved effective, therefore highlighting the potential need for a combination with immune checkpoint inhibitors. Additionally, the TME was altered to be more immunogenic with fewer M2 macrophages and MDSCs.^[^ ^87^ ^]^ IMSA101 in combination with CAR‐T cells showed tumor clearance in an immunogenic model of melanoma and increased overall survival in a less immunogenic model of pancreatic cancer. Notably, an enhanced IL‐18 signature was detected upon combination treatment, which is important for the induction of IFNγ secretion. This suggests that CAR‐T cell efficacy is increased in combination with STING activation.^[^ ^88^ ^]^ IT cGAMP administration together with mesothelin‐targeting CAR‐NK cell treatment improved the survival of Aspc‐1 cell line‐bearing mice.^[^ ^89^ ^]^ Similarly, combining mesothelin‐targeting CAR‐NK cells with the STING agonist ADU‐S100 promoted growth suppression of mesothelin^+^ malignant pleural mesothelioma spheroids and enhanced killing, while the STING agonist had no toxic effect on the NK cells.^[^ ^38^ ^]^ These preclinical findings highlight the potential of STING agonists to turn a “cold” TME into a “hot” one, while also increasing the infiltration of CAR‐T and CAR‐NK cells.

Next to CAR‐transduced cells, TCR‐T cell therapies were explored in combination with a STING agonist as well. A combination of diABZI and TCR‐T cells, which recognize one of the most targeted antigen types, NY‐ESO‐1, enhanced tumor cell apoptosis in vitro and in vivo. This treatment also increased the expression of the cancer cell antigen NY‐ESO and HLA‐A2 presentation as well as TCR activation. Additionally, an elevated expression of IFNγ in TCR engineered T cells was measured,^[^ ^64^ ^]^ highlighting the potential of STING agonists to improve TCR‐T cell therapy

However, clinical trials will be needed to evaluate the safety of these treatment combinations. Furthermore, investigations into whether administering STING agonist treatment in an adjuvant or neo‐adjuvant setting would potentially optimize the efficacy of CAR‐transduced cells or TCR‐T cells. Additionally, to overcome possible antagonistic effects mediated by the STING agonist such as the upregulation of immune checkpoints, it might be worth investigating combinations using CAR cells or TCR‐T cells, STING agonists, and ICIs together with careful consideration of potential side effects.

## 6 Challenges and Limitations of STING Agonists

From the above‐mentioned clinical trials, it is evident that STING agonists have not translated the efficacy observed in preclinical studies into successful outcomes when used as monotherapy. The lack of clinical efficacy of STING‐targeting agents can be attributed to several factors including poor recognition of human STING, limited bioavailability, the need for intratumoral administration, T cell overactivation, NK cell suppression, and immunosuppressive factors. These challenges highlight the complexities of translating STING‐targeted therapies into effective treatments, as discussed further below.

### 6.1 Missing Recognition of Human STING

DMXAA initially showed promising results both in in vitro and in vivo.^[^ ^58^ ^]^ However, DMXAA failed in a clinical phase III study in non‐small‐cell lung cancer patients. As mentioned in section 4, DMXAA could only recognize murine STING.^[^ ^90^ ^]^ Recently, it was demonstrated that DMXAA functions as a partial STING agonist, performing a more suppressive effect on the STING‐IRF3 axis than on the NF‐κB axis.^[^ ^60^ ^]^ Since NC NF‐κB activation through STING has been linked to immunosuppressive effects and mediating tumor growth, this could potentially explain the lack of effectiveness observed in clinical trials. However, research has focused on developing STING agonists capable of recognizing both human and mouse STING to overcome this limitation.

### 6.2 Limited Bioavailability

CDNs are known to have short half‐life (<60 min) such as ADU‐S100. Clinical trial data showed that ADU‐S100 was cleared from the bloodstream in two phases, with the final phase already completed after two hours.^[^ ^61a^ ^]^ Natural CDNs also have poor membrane permeability due to their relatively large size as well as hydrophilicity and high charge, which prevents them from diffusing easily through the membrane. Furthermore, there are no specific transporters for CDNs.^[^ ^56^ ^]^ Additionally, they have a high susceptibility to enzymatic degradation by ENPP1 in the extracellular space,^[^ ^52b^ ^]^ limiting their administration to IT delivery and reducing their effectiveness. As a result, investigations are focusing on the development of more stable compounds to improve their effectiveness and allow systemic administration.

### 6.3 Intratumoral Versus Systemic Administration

Due to the limitation of IT injection for many STING agonists, although this approach provides a direct way to investigate the safety and pharmacodynamics of CDNs, it cannot easily be adapted to all tumor types. This makes many patients ineligible for this treatment. However, IT administration has been shown to be generally safe, alone and in combination with other agents, with potential side effects including pyrexia, chills, pain at the injection site, and increased liver enzymes.^[^ ^61a^ ^]^ To overcome the limitations of IT administration, several systemic STING agonists are under preclinical investigation.^[^ ^61^, ^63^, ^65^ ^]^ However, systemic delivery of such a potent agonist raises other concerns regarding both the efficacy and safety of the applied drug regarding cytokine release syndrome, tissue toxicity, or autoimmunity due to nonspecific delivery. This underscores the importance of safety assessments in clinical trials and the ongoing development of novel delivery platforms.

### 6.4 Overactivation of T cells

Activation of the cGAS‐STING pathway by STING agonists, resulted in overactivation of T cells causing stress and T cell death due to continuous cytokine stimulation.^[^ ^91^ ^]^ While STING treatment with DMXAA enhanced the type I IFN response, it also showed a dose‐dependent effect on T cells in the upregulation of pro‐apoptotic genes, which was not observed with doses below 5 µg ml^−1^.^[^ ^92^ ^]^ This effect appeared to be specific to T cells since it was not detected in other immune cells such as macrophages or DCs when DMXAA was administered at higher doses (10 µg ml^−1^).^[^ ^93^ ^]^ Moreover, this pro‐apoptotic effect on the T cells was not detected when alternate STING agonists were used,^[^ ^38^, ^86^ ^]^ thereby suggesting that the STING‐mediated T cell death is dose‐ and STING agonist dependent. Additionally, STING agonist treatment may induce T cell death by stimulating different extents of calcium leakage from the ER as an innate immune checkpoint.^[^ ^91^ ^]^ These results highlight a limitation in the use of STING agonists together with CAR‐T or TCR‐T cell therapies.

### 6.5 Suppression of NK Cells

Moreover, several studies have raised concerns about a potential NK cell‐suppressing role through STING agonist treatment. Systemic administration of cGAMP, MSA‐2 or diABZI led to increased numbers of regulatory B cells (*B*_regs_) in the TME of PDAC‐bearing mice, demonstrating enhanced secretion of IL‐35, consequently inhibiting NK cell activity.^[^ ^94^ ^]^ Additionally, the systemic treatment with cGAMP alone did not affect tumor growth in comparison to the control group. These results implicate an immunosuppressive function, contradicting the results from Knelson et al. and Da et al., which demonstrated increased NK cell activation after ADU‐S100 treatment^[^ ^38^ ^]^ and augmented CAR‐NK cell treatment efficacy in PDAC when combined with cGAMP.^[^ ^89^ ^]^ While some studies illustrate a positive effect of STING agonists on NK cells and enhanced tumor cell killing upon combined treatment, others reveal a negative influence mediated by *B*_regs_. Discrepancies in results may be attributed to the use of different mouse models, variations in the route of administration or dosage as well as the use of different STING agonists.

### 6.6 Immunosuppressive Factors

Additionally, within the TME, other immunosuppressive factors play a role such as indoleamine 2,3‐dioxygenase 1 (IDO), cyclooxygenase 2 (COX2), or transforming growth factor beta (TGF‐β), which function as negative regulators of the anti‐tumoral immune response. Specifically, IDO inhibits the T cell metabolism, while COX2 mediates apoptotic resistance and proliferation of cancer cells.^[^ ^95^ ^]^ Notably, STING activation has been shown to increase IDO.^[^ ^96^ ^]^ TGF‐β enables cancer cell invasion, therapeutic resistance, and suppression of other immune cells such as T and NK cells.^[^ ^97^ ^]^ *T*_regs_ in multiple myeloma (MM) demonstrated TGF‐β1 secretion, thereby transcriptionally suppressing the mRNA levels of cGAS and STING.^[^ ^98^ ^]^ In spontaneous mammary specific polyomavirus middle T antigen overexpression mouse model (MMTV‐PyMT), TGF‐β limited the IFN‐induced tumor regression.^[^ ^99^ ^]^ Furthermore, STING activation induced the upregulation of PD‐L1 on various immune cells^[^ ^100^ ^]^ like bone marrow‐derived DCs^[^ ^76^ ^]^ and tumor cells.^[^ ^101^ ^]^ Notably, Ghaffari et al. detected elevated levels of PD‐L1 on macrophages and MDSCs, alongside an increased PD‐1 expression on CD8^+^ T cells following STING agonist treatment in ovarian cancer. Consequently, they combined the agonist with an anti‐PD‐1 antibody, highlighting the importance of immune priming to increase treatment response.^[^ ^77^ ^]^ The NC NF‐κB pathway can result in the secretion of tumor‐growth supporting cytokines such as IL‐6. In radiotherapy, the activation of the NC NF‐κB pathway in DCs negatively regulated irradiation‐induced anti‐tumor immunity by controlling the type I IFN expression.^[^ ^26^ ^]^ This underscores a limitation in the immune response during radiotherapy, particularly mediated through the NC NF‐κB pathway. Altogether, these results emphasize the importance of the TME and secreted factors, which can influence the effectiveness of the treatment by modulating the immune response.

### 6.7 Promoting Tumor Growth

All the above‐mentioned results indicate a rather immunosuppressive effect of STING agonists on the tumor. However, it is crucial to consider the different development stages of tumors and the heterogeneity between tumor types. Understanding these factors will be essential for determining potential therapeutic outcomes.

Across the different stages of tumor progression from primary tumor to metastasis, the administration of STING agonists could be either beneficial or further stimulate tumor growth.^[^ ^11^, ^33^ ^]^ In the early stages of tumor development, DNA damage can lead to MN formation, activating the cGAS‐STING pathway. This, in turn, promotes cytokine and chemokine production, enhancing the recruitment of immune cells into the TME.^[^ ^13^, ^15^ ^]^ Nevertheless, DNA from MN can maintain CIN and lead to metastasis.^[^ ^33a^ ^]^ In primary tumors STING activation is mostly associated with the switch of M2 into M1 macrophages,^[^ ^42b^ ^]^ the activation of DCs, and subsequently T cell activation, supporting an anti‐tumoral immune response.^[^ ^14^, ^15^ ^]^ However, it is important to note that in more advanced stages, particularly in the metastatic setting, it becomes increasingly challenging. Here the application of STING agonists could be detrimental, promoting tumorigenesis.^[^ ^102^ ^]^ Additionally, primary tumors often display a CIN^low^ phenotype, while metastasis present a CIN^high^ phenotype. However, primary tumors can also exhibit a CIN^high^ phenotype, relying on a protective role of the inflammatory response, with a CIN‐driven inflammation important for tumor survival and growth.^[^ ^27^ ^]^ Breast cancer cells with a CIN^high^ phenotype demonstrated a higher expression of NC NF‐κB genes, indicating higher treatment resistance. Additionally, breast and lung cancer patients with CIN^high^ tumors were associated with shorter disease‐free survival.^[^ ^33a^ ^]^ Furthermore, in vivo studies using triple‐negative breast cancer cell lines demonstrated that CIN^high^ tumors contained anti‐inflammatory macrophages, granulating MDSCs, and dysfunctional T cells. In contrast, CIN^low^ tumors displayed a more pro‐inflammatory environment with activated DCs and CD4^+^ T helper cells,^[^ ^34^ ^]^ emphasizing the role of the CIN phenotype. Besides, CIN^high^ tumors with chronic STING activation have shown decreased levels of STING in cancer cells.^[^ ^34^ ^]^ Consequently, administering STING agonists may not be ideal for tumors with a CIN^high^ phenotype, further promoting tumor growth.

Apart from the CIN phenotype, there might be an inverse relationship between tumors with high cGAS^+^ MN and low STING protein expression, which correlated with reduced TILs (cGAS^high^STING^low^). In contrast, tumors with low cGAS^+^ MN were associated with higher STING expression and a more favorable diagnosis (cGAS^low^STING^high^).^[^ ^34^ ^]^ Tumors with high STING protein expression demonstrated an upregulation of PD‐1 on CD8^+^ T cells, emphasizing that these tumors might benefit from STING agonists together with checkpoint inhibitors.^[^ ^41^ ^]^ The classification into two groups could help improve treatment strategies, as the last group (cGAS^low^STING^high^) may benefit from STING agonists while the first (cGAS^high^STING^low^) could respond more effectively to STING inhibitors.^[^ ^34^, ^103^ ^]^ In addition, a high cGAS micronuclear burden was associated with increased myeloid cell presence and poorer prognosis in esophageal cancer, indicating that myeloid‐driven inflammation contributes to tumor progression.^[^ ^51^ ^]^ STING protein expression in both tumor and endothelial cells was crucial for inducing an effective anti‐tumor response when treated with cGAMP monotherapy in B16‐F10 tumors. However, in 4T1 breast cancer tumors, the response to cGAMP was weaker, indicating a tumor‐type‐specific difference in STING pathway activation.^[^ ^51^, ^56^ ^]^ In the B16‐F10 model, it was demonstrated that STING expression in non‐tumor cells was needed to mediate the anti‐tumor effect.^[^ ^104^ ^]^ This emphasizes the role of the TME in responding to STING agonist treatment. High STING expression has been observed in several cancers, including breast cancer, clear renal cell carcinoma, colorectal adenocarcinoma, hepatocellular carcinoma, and papillary carcinoma of the thyroid. This expression was linked to a more aggressive cancer phenotype and increased PD‐L1 levels 0.1 cancer cells.^[^ ^105^ ^]^

Moreover, repetitive stimulations with STING agonists induced IFN tachyphylaxis, a phenomenon, where repetitive IFN stimulation reduces the response, suggesting that prolonged exposure to STING agonists leads to reduced efficacy due to immune system desensitization.^[^ ^34^ ^]^

These data highlight the importance of considering cGAS and STING expression within the TME, as well as the CIN phenotype when modifying treatments for patients. It also highlights a context‐dependent pro‐tumorigenic effect of STING activation. Further research is required to determine the effectiveness of STING agonist monotherapy in tumors with low immunogenicity or low immune cell count.

## 7 Overcoming the Challenges and Limitations of STING Agonists

### 7.1 Potential Strategies to Improve Administration

To overcome this limitation of using free agonists and their intratumoral administration, research interest has shifted towards the encapsulation of agonists in nanocarriers or the use of antibody‐drug conjugates (ADC). Nanocarriers include liposomes, polymersomes, peptide nanodrugs, or even metal‐based nanoparticles and they allow specific systemic delivery of compounds into the tumor bed without the need for IT administration.^[^ ^106^ ^]^ Namely, the use of a biopolymer containing a STING agonist (cdGMP) and NKG2D‐CAR‐T cells was placed directly on a mouse pancreatic tumor, effectively delivering CAR‐T cells into the tumor bed to eradicate tumor cells. Furthermore, the STING agonist was released over time at the tumor site, transforming the tumor bed into a “self‐vaccine site”.^[^ ^107^ ^]^ Nevertheless, the device needs to be implanted in combination with cytoreductive surgery and additional research is required to investigate the impact on metastasis. However, this approach provides a solution to deliver the product to its intended destination. Similarly, demonstrated encapsulated cGAMP and CpG ODN plus the model antigen OVA an induction of a boosted Th1 immune response in vitro, decreased M2 macrophages, and reduced tumor volume of up to 70% in melanoma in vivo.^[^ ^108^ ^]^ The use of a bridging‐lipid nanoparticle targeting CD47/PD‐1 on tumor‐associated myeloid cells in glioblastoma, combined with diABZI, reprogrammed these cells from an immunosuppressive into an anti‐tumoral state.^[^ ^109^ ^]^ Furthermore, Nakamura et al. demonstrated that a STING LNP enhanced NK cell activity in a B16‐F10 melanoma model and showed that the enhanced tumor control was mediated by NK cells rather than CD8^+^ T cells.^[^ ^110^ ^]^ Using nanocarriers to deliver STING agonists can improve the half‐life as well as bioavailability, especially if natural CDNs are used. Nevertheless, these strategies cannot avoid the death of immune cells in the TME due to overactivation by the STING agonist, even though this effect is less pronounced compared to using free agonists.^[^ ^106a^ ^]^

ADCs present another novel approach to overcome the side effects associated with systemic STING agonist administration. Multiple STING agonist ADCs with different targets induced IFNs 100‐fold more potently and enhanced tumor cell killing relative to free agonists in vitro. In vivo results showed in two different tumor models durable responses at lower doses compared to diABZI with increased tumor‐localized inflammatory cytokines.^[^ ^111^ ^]^ The cGAMP analogue IMSA172 conjugated to an anti‐EGFR binding antibody led to suppression of B16‐F10 tumor growth dependent on the expression of EGFR. Furthermore, the study demonstrated increased DC, T cell, and NK cell activation and repolarization of M2 macrophages.^[^ ^112^ ^]^ After promising in vivo data, the ADC XMT‐2056 binding HER2 entered a clinical phase I trial (NCT05514717).^[^ ^113^ ^]^ A CD11b‐STINGa‐ADC demonstrated STING activation in tumor cells and complete tumor regression in an ovarian and breast cancer xenograft mouse model. Importantly, this STINGa‐ADC outperformed the free, systemic STING agonist diABZI at a 50‐fold lower dose and induced much lower serum cytokine elevations over time. Additionally, the primary driver of the anti‐tumor effect of STINGa‐ADC is the innate immune response, triggered downstream of STING activation, as observed in tumor xenograft SCID mice.^[^ ^114^ ^]^

In conclusion, both IT and systemic delivery have their benefits and limitations, and more studies regarding toxicities and side effects of nanocarriers, ADCs, and systemic STING agonists are needed.

### 7.2 How to Overcome T cell Overactivation and NK Cell Inhibition

**Figure 4:**
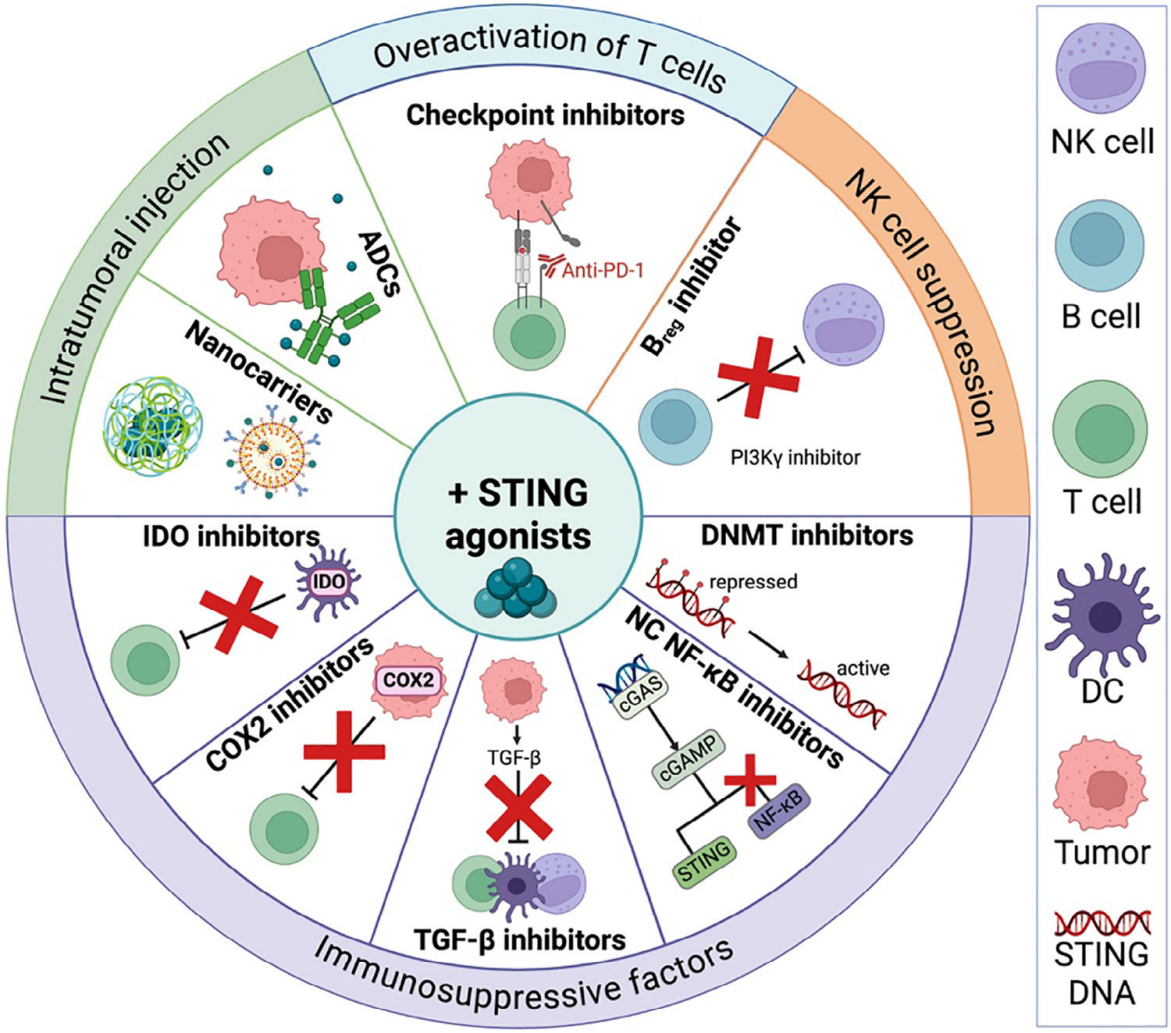
Potential combination strategies for STING agonists to combat potentially immunosuppressive effects. To counteract T cell overactivation, checkpoint inhibitors can be used, while PI3Kγ inhibitors may alleviate NK cell suppression induced by the STING agonist. The combination of DNA methylase (DNMT) inhibitors and STING agonists can help to reverse tumor‐mediated STING silencing. Inhibiting the non‐canonical (NC) nuclear factor kappa B (NF‐κB) pathway together with a STING agonist may enhance the anti‐tumoral type I interferon response. The use of transforming growth factor beta (TGF‐β) inhibitors can reverse immune suppression on immune cells induced by STING activation in the tumor microenvironment. To target T cell inhibition in the TME, cyclooxygenase 2 (COX2) and indoleamine 2,3‐dioxygenase (IDO) inhibitors can be useful in combination with STING agonists. Nanocarriers and antibody‐dependent conjugates (ADCs) can deliver STING agonists in a systemic setting directly to the tumor, overcoming the need for intratumoral injection. Figure created with BioRender.

To prevent T cell toxicity, Knelson et al. propose to use a burst‐dose agonism, e.g. using a high dose of the STING agonist first to strongly activate the immune system. This approach could delay T cells from attacking too soon due to overactivation. This method allows NK cells and DCs to be activated first, which then cross‐prime the T cells, thereby potentially enhancing the overall immune response.^[^ ^38^ ^]^ Another strategy to prevent T cell toxicity involves pre‐activation of T cells prior to administration. Besides, the nature and the potency of the STING agonist dose influence the induction of T cell death; lower doses have been demonstrated to not induce this effect, therefore highlighting the importance of careful dose selection. The timing of administration may potentially influence the results as well. One perspective suggests that the therapeutic impact occurs in two distinct phases. In the initial phase (within the first 24 h), damage to the tumor vasculature is thought to occur through STING‐mediated apoptosis and activation of innate immune cells which primarily produce type I IFNs. Subsequently, a second phase then leads to T cell activation due to activated APCs.^[^ ^11d^ ^]^ Considering this scheme, administering the STING agonist first and the ICI treatment afterward could circumvent the immunosuppressive effects associated with STING agonists. Since STING agonists have been shown to increase the expression of immune checkpoints on T cells such as PD‐1, a combination with CPIs could be beneficial (as discussed in section 5.2.1)^[^ ^77^, ^79^ ^]^ (**Figure** **4**). However, it is important to mention that PD‐1 expression on CD8^+^ T cells induced by STING agonists depends on the tumor model and TME.^[^ ^41^ ^]^

To overcome NK cell suppression, in a systemic delivery setting, a combination of a STING agonist with a PI3Kγ inhibitor reduced the number of *B*_regs_ in PDAC while increasing the number of myeloid cells, thereby mediating tumor control.^[^ ^115^ ^]^ This highlights the potential of *B*_reg_ inhibitors together with CAR‐NK cell therapy (Figure 4).

### 7.3 How to Counteract Immunosuppressive Factors

As mentioned earlier, IDO is a great immune cell inhibitor in the TME. In colorectal cancer‐bearing mice, the STING agonist diABZI decreased tumor volume and increased survival when combined with the IDO inhibitor 1‐MT. This combination further increased DC and CD8^+^ T cell numbers while reducing MDSCs compared to STING agonist monotherapy.^[^ ^116^ ^]^ These findings indicate that the IDO inhibitor might rescue the induction of MDSCs by STING agonist treatment. However, the use of an IDO‐blocking antibody together with a STING agonist (ADU‐S100) did not show a significant difference in mouse survival in MC38 tumors. Conversely, combining the STING agonist with a COX‐2 inhibitor led to a significant reduction in tumor volume and improved survival in a mouse model of colon cancer.^[^ ^42c^ ^]^ The survival of the mice was further extended when an anti‐PD‐1 antibody was added to the combination. To counteract the immune suppression mediated by TGF‐β, a neutralizing antibody together with a STING agonist reduced the immune suppressive effect and suppressed *T*_regs_ in an multiple myeloma mouse model.^[^ ^98^ ^]^ In spontaneous MMTV‐PyMT mammary tumors blocking of TGF‐β restored the activity of DMXAA and therefore the IFN‐induced tumor regression^[^ ^99^ ^]^ (Figure 4).

To counteract the expression of immune checkpoints within the TME, the use of a STING agonist (IMSA172) conjugated to an anti‐epidermal growth factor receptor (EGFR) antibody increased PD‐L1 on DCs within the tumor and tumor‐draining lymph nodes, while the most pronounced anti‐tumor effect was seen in combination with an anti‐PD‐L1 antibody in B16‐F10 bearing mice.^[^ ^112^ ^]^ Importantly, PD‐L1 expression is not exclusively upregulated on immune cells but tumor cells as well, such as for liver cancer^[^ ^117^ ^]^ or non‐small cell lung cancer.^[^ ^118^ ^]^ Additionally, the combination of MSA‐2 with an anti‐TGF‐βR2/PD‐L1 bispecific antibody promoted DC maturation, naïve T cell activation, and enhanced NK cells in different cancer mouse models (melanoma, breast, or colon cancer). By targeting both TGF‐β and PD‐L1 simultaneously with the STING agonist, even immune‐excluded or immune‐deserted tumors could benefit therapeutically.^[^ ^76^ ^]^ To further improve the anti‐tumor response, NC NF‐kB inhibition, but not canonical NF‐kB inhibition promoted tumor regression in combination with irradiation^[^ ^26^ ^]^ (Figure 4). To enhance STING expression within the TME, the use of DNA methyltransferase (DNMT) inhibitors can improve the response. The use of KDM5 inhibitors in human papilloma virus positive (HPV^+^) head and neck or cervical tumors restored STING expression and induced an anti‐tumor response^[^ ^49^ ^]^ (Figure 4). A combination of the DNMT inhibitor 5AZADC and ADU‐S100 restored the tumor‐cell intrinsic STING signaling defect due to epigenetic programming and enhanced the anti‐tumor response against melanoma in vivo.^[^ ^50^ ^]^ For CIN^high^ tumors, the STING inhibitor C‐176 led to the downregulation of pathways related to inflammation, EMT, and ER stress and prolonged the survival of CIN^high^ tumor‐bearing mice.^[^ ^34^ ^]^ Using tocilizumab, blocking the IL‐6 receptor, the IL‐6 mediated immune suppression was prevented. In addition, the growth of triple‐negative breast cancer cells was impaired, and the outgrowth of CIN^+^ cells was delayed.^[^ ^27^ ^]^

**Figure 5:**
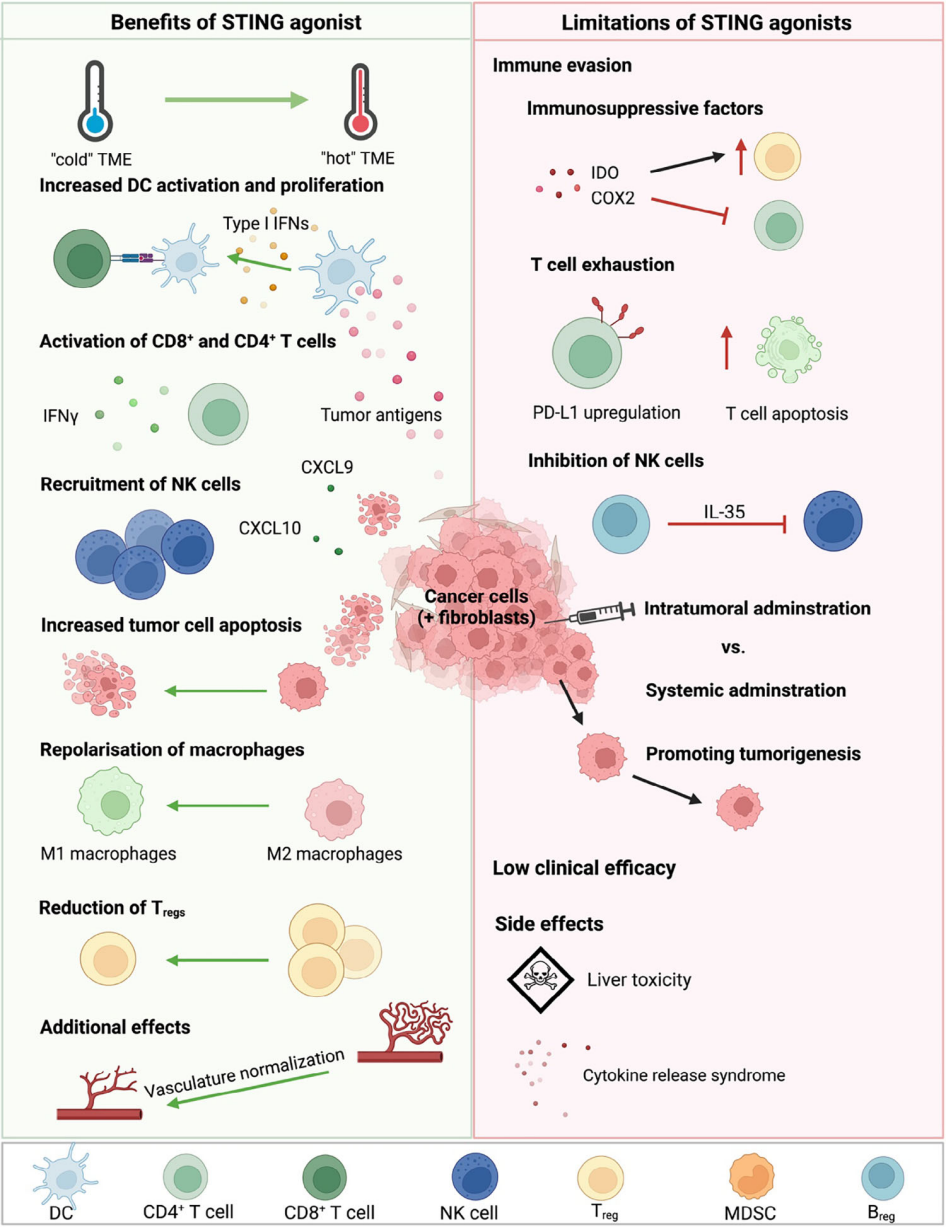
The benefits and limitations of STING agonists for cancer immunotherapy. STING agonists can transform a pro‐tumoral “cold” tumor microenvironment (TME) into a “hot” immunogenic TME through the activation of antigen‐presenting cells (APCs) via type I interferons (IFNs) resulting from STING activation. This facilitates the activation of CD4^+^ and CD8^+^ T cells, which subsequently infiltrate the TME, leading to increased cancer cell death. Moreover, STING agonists have been shown to promote the infiltration of natural killer (NK) cells and the turnover of M2 pro‐tumoral macrophages into M1 anti‐tumoral macrophages, the reduction of regulatory T cells and normalize the vasculature. However, STING agonists can also elevate immunosuppressive factors, augmenting PD‐L1 expression, driving T cell exhaustion, promoting the differentiation of myeloid‐derived suppressor cells, and raising levels of regulatory B cells (*B*_regs_), which may lead to clinical side effects such as cytokine release syndrome or liver toxicity. Green arrows indicate positive effects and red arrows indicate negative effects. Figure created with BioRender.

Therefore, to counteract undesirable side effects mediated by STING agonists, combination strategies with IDO, TGF‐β, DNMT, NC NF‐kB, *B_reg_* or COX2 inhibitors may be necessary to improve clinical outcomes. To summarize, the use of STING agonists faces significant challenges from immune‐related adverse events to the complexity of determining TME, CIN phenotype, timing, dosing, and the choice of STING agonist (**Figure** **5**). To address these disadvantages and optimize the clinical utilization of STING agonists, further research is necessary.

## 8 Future Perspectives and Concluding Remarks

In the past years, immunotherapy has gained significant attention, aiming to utilize the patient's own immune system in the fight against cancer and to amplify the anti‐tumoral immune response. The cGAS/STING pathway works as a potent regulator of the anti‐tumoral immune response. Since the discovery of the first STING agonist, DMXAA, various agonists have been developed with the goal of improving bioavailability, mitigate toxicity, and refining routes of administration. While multiple preclinical studies have shown increased anti‐tumor efficacy of CD8^+^ T cells and NK cells as well as increased tumor control after agonist treatment, negative side effects such as T cell toxicity, inhibition of NK cells by IL‐35‐producing *B*_regs_ as well as challenges with the route of administration even promoting tumor growth, have been observed. Future research directions should include a deeper analysis of the impact of STING agonists on cells with low or no STING as well as improving our understanding of the mechanisms underlying STING activation in the TME. While single‐agent therapy might suffice, investigating potential combination strategies such as anti‐PD‐1 or anti‐PD‐L1 antibodies, IDO, DNMT, or TGF‐β inhibitors as well as *B_reg_* inhibitors, holds promise to enhance the effect of STING agonist treatment in the clinic and to address immunomodulatory mechanisms. In such regard, it is evident that the most efficient way forward will consist of a combination strategy with other immune therapies.

In summary, treatment efficacy depends on the choice of agonist tailored to the cancer type. While certain STING agonists exhibit promising responses at specific doses and certain cancer types, others may yield less favorable outcomes. Furthermore, more clinical data is needed, particularly for a comprehensive understanding of immunosuppressive effects in humans.

## Conflict of Interest

The authors declare no conflict of interest.

## Author Contributions

Conceptualization is performed by L.G, C.D, E.S., and J.RM.V.A; writing – original draft preparation is performed by L.G; writing – review and editing is performed by L.G, C.D, E.S., and J.RM.V.A; visualization is performed by L.G.; supervision is performed by C.D, E.S., and J.RM.V.A; funding acquisition is provided by E.S. and J.RM.V.A. All authors have read and agreed to the published version of the manuscript.

## Data Availability

The dataset analyzed in Figure 3 is available in Pubmed using the terms “STING agonists” and “cancer” focusing on the years 2014 to 2024 and are available under the following URL: https://pubmed.ncbi.nlm.nih.gov/?term=%28STING+agonists%29+AND+%28Cancer%29&filter=years.2014‐2023.

## References

[1] W. H. Organization , World Health Organization 2024, Retrieved from, https://www.who.int/news/item/01‐02‐2024‐global‐cancer‐burden‐growing‐amidst‐mounting‐need‐for‐sevices.

[2] Y. Shiravand , F. Khodadadi , S. M. A. Kashani , S. R. Hosseini‐Fard , S. Hosseini , H. Sadeghirad , R. Ladwa , K. O'Byrne , A. Kulasinghe , Curr. Oncol. 2022, 29, 3044. 35621637 10.3390/curroncol29050247 PMC9139602

[3] a) M. S. Carlino , J. Larkin , G. V. Long , Lancet 2021, 398, 1002; 34509219 10.1016/S0140-6736(21)01206-X

[4] a) R. J. Motzer , B. Escudier , D. F. McDermott , S. George , H. J. Hammers , S. Srinivas , S. S. Tykodi , J. A. Sosman , G. Procopio , E. R. Plimack , D. Castellano , T. K. Choueiri , H. Gurney , F. Donskov , P. Bono , J. Wagstaff , T. C. Gauler , T. Ueda , Y. Tomita , F. A. Schutz , C. Kollmannsberger , J. Larkin , A. Ravaud , J. S. Simon , L.‐A. Xu , I. M. Waxman , P. Sharma , N. Engl. J. Med. 2015, 373, 1803; 26406148 10.1056/NEJMoa1510665 PMC5719487

[5] K. Onoi , Y. Chihara , J. Uchino , T. Shimamoto , Y. Morimoto , M. Iwasaku , Y. Kaneko , T. Yamada , K. Takayama , J Clin. Med. 2020, 9,1362 32384677 10.3390/jcm9051362 PMC7290914

[6] a) G. K. Abou‐Alfa , G. Lau , M. Kudo , S. L. Chan , R. K. Kelley , J. Furuse , W. Sukeepaisarnjaroen , Y.‐K. Kang , T. V. Dao , E. N. D. Toni , L. Rimassa , V. Breder , A. Vasilyev , A. Heurgué , V. C. Tam , K. Mody , S. C. Thungappa , Y. Ostapenko , T. Yau , S. Azevedo , M. Varela , A.‐L. Cheng , S. Qin , P. R. Galle , S. Ali , M. Marcovitz , M. Makowsky , P. He , J. F. Kurland , A. Negro , et al., NEJM Evidence 2022, 1, EVIDoa2100070; 38319892 10.1056/EVIDoa2100070

[7] E. M. O'Reilly , D.‐Y. Oh , N. Dhani , D. J. Renouf , M. A. Lee , W. Sun , G. Fisher , A. Hezel , S.‐C. Chang , G. Vlahovic , O. Takahashi , Y. Yang , D. Fitts , P. A. Philip , JAMA Oncology 2019, 5, 1431. 31318392 10.1001/jamaoncol.2019.1588 PMC6647002

[8] a) A. R. Haas , J. L. Tanyi , M. H. O'Hara , W. L. Gladney , S. F. Lacey , D. A. Torigian , M. C. Soulen , L. Tian , M. McGarvey , A. M. Nelson , C. S. Farabaugh , E. Moon , B. L. Levine , J. J. Melenhorst , G. Plesa , C. H. June , S. M. Albelda , G. L. Beatty , Mol. Ther. 2019, 27, 1919; 31420241 10.1016/j.ymthe.2019.07.015 PMC6838875

[9] P. Guha , K. R. Heatherton , K. P. O'Connell , I. S. Alexander , S. C. Katz , Biomedicines 2022, 10, 655. 35327456 10.3390/biomedicines10030655 PMC8945484

[10] I. Mellman , D. S. Chen , T. Powles , S. J. Turley , Immunity 2023, 56, 2188. 37820582 10.1016/j.immuni.2023.09.011

[11] a) R. H. Vonderheide , Annu. Rev. Med. 2020, 71, 47; 31412220 10.1146/annurev-med-062518-045435

[12] R. Yu , B. Zhu , D. Chen , Cell. Mol. Life Sci. 2022, 79, 191. 35292881 10.1007/s00018-022-04219-z PMC8924142

[13] Z. Liu , D. Wang , J. Zhang , P. Xiang , Z. Zeng , W. Xiong , L. Shi , Cancer Lett. 2023, 577, 216409. 37748723 10.1016/j.canlet.2023.216409

[14] I. Benoit‐Lizon , E. Jacquin , T. R. Vargas , C. Richard , A. Roussey , L. D. Zuffo , T. Martin , A. Melis , D. Vinokurova , S. H. Shahoei , A. B. Garcia , C. Pignol , S. Giorgiutti , R. Carapito , R. Boidot , F. Végran , R. A. Flavell , B. Ryffel , E. R. Nelson , P. Soulas‐Sprauel , T. Lawrence , L. Apetoh , J. ImmunoTherapy Cancer 2022, 10, 003459. 10.1136/jitc-2021-003459 PMC8804688 35091453

[15] a) I. Škrnjug , C. A. Guzmán , C. Ruecker , PLoS One 2014, 9, 110150; 10.1371/journal.pone.0110150 PMC4190368 25295996

[16] a) O. Demaria , A. De Gassart , S. Coso , N. Gestermann , J. Di Domizio , L. Flatz , O. Gaide , O. Michielin , P. Hwu , T. V. Petrova , F. Martinon , R. L. Modlin , D. E. Speiser , M. Gilliet , Proc. Natl. Acad. Sci. USA 2015, 112, 15408; 26607445 10.1073/pnas.1512832112 PMC4687570

[17] D. S. Chen , I. Mellman , Immunity 2013, 1, 10.1016/j.immuni.2013.07.012. 23890059

[18] K. R. B. Lanng , E. L. Lauridsen , M. R. Jakobsen , Nat. Immunol. 2024, 25, 1144. 38918609 10.1038/s41590-024-01872-3

[19] L. Sun , J. Wu , F. Du , X. Chen , Z. J. Chen , Science 2013, 339, 786. 23258413 10.1126/science.1232458 PMC3863629

[20] a) H. E. Volkman , S. Cambier , E. E. Gray , D. B. Stetson , Elife 2019, 8, 1; 10.7554/eLife.47491 PMC6927687 31808743

[21] J. Wu , L. Sun , X. Chen , F. Du , H. Shi , C. Chen , Z. J. Chen , Science 2013, 339, 826. 23258412 10.1126/science.1229963 PMC3855410

[22] N. Dobbs , N. Burnaevskiy , D. Chen , V. K. Gonugunta , N. M. Alto , N. Yan , Cell Host Microbe 2015, 18, 157. 26235147 10.1016/j.chom.2015.07.001 PMC4537353

[23] Y. Zhu , X. An , X. Zhang , Y. Qiao , T. Zheng , X. Li , Mol. Cancer 2019, 18, 152. 31679519 10.1186/s12943-019-1087-y PMC6827255

[24] S. L. Pogue , B. T. Preston , J. Stalder , C. R. Bebbington , P. M. Cardarelli , J. Interferon Cytokine Res. 2004, 24, 131. 14980077 10.1089/107999004322813372

[25] S.‐R. Woo , M. B. Fuertes , L. Corrales , S. Spranger , M. J. Furdyna , M. Y. K. Leung , R. Duggan , Y. Wang , G. N. Barber , K. A. Fitzgerald , M.‐L. Alegre , T. F. Gajewski , Immunity 2014, 41, 830. 25517615 10.1016/j.immuni.2014.10.017 PMC4384884

[26] Y. Hou , H. Liang , E. Rao , W. Zheng , X. Huang , L. Deng , Y. Zhang , X. Yu , M. Xu , H. Mauceri , A. Arina , R. R. Weichselbaum , Y. X. Fu , Immunity 2018, 49, 490. 30170810 10.1016/j.immuni.2018.07.008 PMC6775781

[27] C. Hong , M. Schubert , A. E. Tijhuis , M. Requesens , M. Roorda , A. van den Brink , L. A. Ruiz , P. L. Bakker , T. van der Sluis , W. Pieters , M. Chen , R. Wardenaar , B. van der Vegt , D. C. J. Spierings , M. de Bruyn , M. A. T. M. van Vugt , F. Foijer , Nature 2022, 607, 366. 35705809 10.1038/s41586-022-04847-2

[28] K. R. Balka , C. Louis , T. L. Saunders , A. M. Smith , D. J. Calleja , D. B. D'Silva , F. Moghaddas , M. Tailler , K. E. Lawlor , Y. Zhan , C. J. Burns , I. P. Wicks , J. J. Miner , B. T. Kile , S. L. Masters , D. De Nardo , Cell Rep. 2020, 31, 107492. 32268090 10.1016/j.celrep.2020.03.056

[29] S. Yum , M. Li , Y. Fang , Z. J. Chen , Proc. Natl. Acad. Sci. USA 2021, 118, 2100225118. 10.1073/pnas.2100225118 PMC8040795 33785602

[30] J. A. Carozza , V. Bohnert , K. C. Nguyen , G. Skariah , K. E. Shaw , J. A. Brown , M. Rafat , R. von Eyben , E. E. Graves , J. S. Glenn , M. Smith , L. Li , Nat. Cancer 2020, 1, 184. 33768207 10.1038/s43018-020-0028-4 PMC7990037

[31] A. Ablasser , J. L. Schmid‐Burgk , I. Hemmerling , G. L. Horvath , T. Schmidt , E. Latz , V. Hornung , Nature 2013, 503, 530. 24077100 10.1038/nature12640 PMC4142317

[32] H. T. W. Blest , L. Chauveau , Front. Immunol. 2023, 14, 1150705. 37287967 10.3389/fimmu.2023.1150705 PMC10242147

[33] a) S. F. Bakhoum , B. Ngo , A. M. Laughney , J.‐A. Cavallo , C. J. Murphy , P. Ly , P. Shah , R. K. Sriram , T. B. K. Watkins , N. K. Taunk , M. Duran , C. Pauli , C. Shaw , K. Chadalavada , V. K. Rajasekhar , G. Genovese , S. Venkatesan , N. J. Birkbak , N. McGranahan , M. Lundquist , Q. LaPlant , J. H. Healey , O. Elemento , C. H. Chung , N. Y. Lee , M. Imielenski , G. Nanjangud , D. Pe'er , D. W. Cleveland , S. N. Powell , et al., Nature 2018, 553, 467; 29342134 10.1038/nature25432 PMC5785464

[34] J. Li , M. J. Hubisz , E. M. Earlie , M. A. Duran , C. Hong , A. A. Varela , E. Lettera , M. Deyell , B. Tavora , J. J. Havel , S. M. Phyu , A. D. Amin , K. Budre , E. Kamiya , J.‐A. Cavallo , C. Garris , S. Powell , J. S. Reis‐Filho , H. Wen , S. Bettigole , A. J. Khan , B. Izar , E. E. Parkes , A. M. Laughney , S. F. Bakhoum , Nature 2023, 620, 1080. 37612508 10.1038/s41586-023-06464-z PMC10468402

[35] a) K. J. Mackenzie , P. Carroll , C.‐A. Martin , O. Murina , A. Fluteau , D. J. Simpson , N. Olova , H. Sutcliffe , J. K. Rainger , A. Leitch , R. T. Osborn , A. P. Wheeler , M. Nowotny , N. Gilbert , T. Chandra , M. A. M. Reijns , A. P. Jackson , Nature 2017, 548, 461; 28738408 10.1038/nature23449 PMC5870830

[36] D. Jeltema , K. Abbott , N. Yan , J. Exp. Med. 2023, 220, 20220990. 10.1084/jem.20220990 PMC9930166 36705629

[37] A. Marcus , A. J. Mao , M. Lensink‐Vasan , L. Wang , R. E. Vance , D. H. Raulet , Immunity 2018, 49, 754. 30332631 10.1016/j.immuni.2018.09.016 PMC6488306

[38] E. H. Knelson , E. V. Ivanova , M. Tarannum , M. Campisi , P. H. Lizotte , M. A. Booker , I. Ozgenc , M. Noureddine , B. Meisenheimer , M. Chen , B. Piel , N. Spicer , B. Obua , C. M. Messier , E. Shannon , N. R. Mahadevan , T. Tani , P. J. Schol , A. M. Lee‐Hassett , A. Zlota , H. V. Vo , M. Ha , A. A. Bertram , S. Han , T. C. Thai , C. E. Gustafson , K. Venugopal , T. J. Haggerty , T. P. Albertson , A. V. Hartley , et al., Cancer Immunol. Res. 2022, 10, 947. 35678717 10.1158/2326-6066.CIR-22-0017 PMC9357206

[39] K. Takashima , Y. Takeda , H. Oshiumi , H. Shime , M. Okabe , M. Ikawa , M. Matsumoto , T. Seya , Biochem. Biophys. Res. Commun. 2016, 478, 1764. 27608599 10.1016/j.bbrc.2016.09.021

[40] N. K. Wolf , C. Blaj , L. K. Picton , G. Snyder , L. Zhang , C. J. Nicolai , C. O. Ndubaku , S. M. McWhirter , K. C. Garcia , D. H. Raulet , Proc. Natl. Acad. Sci. USA 2022, 119, 2200568119. 10.1073/pnas.2200568119 PMC9295797 35588144

[41] J. Czapla , A. Drzyzga , S. Matuszczak , T. Cichoń , M. Rusin , M. Jarosz‐Biej , E. Pilny , R. Smolarczyk , Front. Oncol. 2023, 13, 1249524. 37655095 10.3389/fonc.2023.1249524 PMC10465696

[42] a) T. Ohkuri , A. Kosaka , K. Ishibashi , T. Kumai , Y. Hirata , K. Ohara , T. Nagato , K. Oikawa , N. Aoki , Y. Harabuchi , E. Celis , H. Kobayashi , Cancer Immunol., Immunother. 2017, 66, 705; 28243692 10.1007/s00262-017-1975-1 PMC11028681

[43] M. Campisi , S. K. Sundararaman , S. E. Shelton , E. H. Knelson , N. R. Mahadevan , R. Yoshida , T. Tani , E. Ivanova , I. Cañadas , T. Osaki , S. W. L. Lee , T. Thai , S. Han , B. P. Piel , S. Gilhooley , C. P. Paweletz , V. Chiono , R. D. Kamm , S. Kitajima , D. A. Barbie , Front. Immunol. 2020, 11, 2090. 33013881 10.3389/fimmu.2020.02090 PMC7507350

[44] J. Czapla , A. Drzyzga , J. Ciepła , S. Matuszczak , M. Jarosz‐Biej , E. Pilny , T. Cichoń , R. Smolarczyk , Cancer Immunol., Immunother. 2024, 73, 148. 38832958 10.1007/s00262-024-03732-3 PMC11150340

[45] G. Leuzzi , A. Vasciaveo , A. Taglialatela , X. Chen , T. M. Firestone , A. R. Hickman , W. Mao , T. Thakar , A. Vaitsiankova , J.‐W. Huang , R. Cuella‐Martin , S. B. Hayward , J. S. Kesner , A. Ghasemzadeh , T. S. Nambiar , P. Ho , A. Rialdi , M. Hebrard , Y. Li , J. Gao , S. Gopinath , O. A. Adeleke , B. J. Venters , C. G. Drake , R. Baer , B. Izar , E. Guccione , M.‐C. Keogh , R. Guerois , L. Sun , et al., Cell 2024, 187, 861. 38301646 10.1016/j.cell.2024.01.008 PMC10980358

[46] C. Li , Q. Shen , P. Zhang , T. Wang , W. Liu , R. Li , X. Ma , X. Zeng , Y. Yin , K. Tao , J. Exp. Clin. Cancer Res. 2021, 40, 315. 34625086 10.1186/s13046-021-02120-4 PMC8501558

[47] M. Ghosh , S. Saha , J. Li , D. C. Montrose , L. A. Martinez , Mol. Cell 2023, 83, 266. 36638783 10.1016/j.molcel.2022.12.023 PMC9993620

[48] M. Ghosh , S. Saha , J. Bettke , R. Nagar , A. Parrales , T. Iwakuma , A. W. M. van der Velden , L. A. Martinez , Cancer Cell 2021, 39, 494. 33545063 10.1016/j.ccell.2021.01.003 PMC8044023

[49] L. Wu , J. Cao , W. L. Cai , S. M. Lang , J. R. Horton , D. J. Jansen , Z. Z. Liu , J. F. Chen , M. Zhang , B. T. Mott , K. Pohida , G. Rai , S. C. Kales , M. J. Henderson , X. Hu , A. Jadhav , D. J. Maloney , A. Simeonov , S. Zhu , A. Iwasaki , M. D. Hall , X. Cheng , G. S. Shadel , Q. Yan , PLoS Biol. 2018, 16, 2006134. 10.1371/journal.pbio.2006134 PMC6095604 30080846

[50] R. Falahat , A. Berglund , P. Perez‐Villarroel , R. M. Putney , I. Hamaidi , S. Kim , S. Pilon‐Thomas , G. N. Barber , J. J. Mule , Nat. Commun. 2023, 14, 1573. 36949064 10.1038/s41467-023-37217-1 PMC10033671

[51] B. Beernaert , E. Erdal , R. Clark , T. Liu , E. M. Hammond , E. E. Parkes , Cancer Res. 2024, 84, 2704.

[52] a) S. Wang , V. Böhnert , A. J. Joseph , V. Sudaryo , J. Swinderman , F. B. Yu , X. Lyu , G. Skariah , V. Subramanyam , L. A. Gilbert , H. Goodarzi , L. Li , (Preprint), bioRxiv 2023.06.01.543353, submitted June 2023; 10.1073/pnas.2313693120 PMC10756298 38117852

[53] J. Li , M. A. Duran , N. Dhanota , W. K. Chatila , S. E. Bettigole , J. Kwon , R. K. Sriram , M. P. Humphries , M. Salto‐Tellez , J. A. James , M. G. Hanna , J. C. Melms , S. Vallabhaneni , K. Litchfield , I. Usaite , D. Biswas , R. Bareja , H. W. Li , M. L. Martin , P. Dorsaint , J.‐A. Cavallo , P. Li , C. Pauli , L. Gottesdiener , B. J. DiPardo , T. J. Hollmann , T. Merghoub , H. Y. Wen , J. S. Reis‐Filho , N. Riaz , et al., Cancer Discovery 2021, 11, 1212. 33372007 10.1158/2159-8290.CD-20-0387 PMC8102348

[54] S. F. Bakhoum , L. C. Cantley , Cell 2018, 174, 1347. 30193109 10.1016/j.cell.2018.08.027 PMC6136429

[55] M. Smith , D. Chin , S. Chan , S. Mahady , L. Campion , C. Morgan , S. Patel , G. Chu , A. Hughes , G. Bignan , P. Connolly , S. Emanuel , K. Packman , L. L. Luistro , Cancer Res. 2020, 80, 5567.

[56] R. Huang , Q. Ning , J. Zhao , X. Zhao , L. Zeng , Y. Yi , S. Tang , Int. Immunopharmacol. 2022, 113, 109304. 36252492 10.1016/j.intimp.2022.109304

[57] L. M. Ching , Z. Cao , C. Kieda , S. Zwain , M. B. Jameson , B. C. Baguley , Br. J. Cancer 2002, 86, 1937. 12085190 10.1038/sj.bjc.6600368 PMC2375421

[58] W. Jing , D. McAllister , E. P. Vonderhaar , K. Palen , M. J. Riese , J. Gershan , B. D. Johnson , M. B. Dwinell , J Immunother. Cancer 2019, 7, 115. 31036082 10.1186/s40425-019-0573-5 PMC6489306

[59] J. Conlon , D. L. Burdette , S. Sharma , N. Bhat , M. Thompson , Z. Jiang , V. A. Rathinam , B. Monks , T. Jin , T. S. Xiao , S. N. Vogel , R. E. Vance , K. A. Fitzgerald , J. Immunol. 2013, 190, 5216. 23585680 10.4049/jimmunol.1300097 PMC3647383

[60] B. Temizoz , T. Shibahara , K. Hioki , T. Hayashi , K. Kobiyama , M. S. J. Lee , N. Surucu , E. Sag , A. Kumanogoh , M. Yamamoto , M. Gursel , S. Ozen , E. Kuroda , C. Coban , K. J. Ishii , Front. Immunol. 2024, 15, 1353336. 38533502 10.3389/fimmu.2024.1353336 PMC10963404

[61] a) F. Meric‐Bernstam , R. F. Sweis , F. S. Hodi , W. A. Messersmith , R. H. I. Andtbacka , M. Ingham , N. Lewis , X. Chen , M. Pelletier , X. Chen , J. Wu , S. M. McWhirter , T. Muller , N. Nair , J. J. Luke , Clin. Cancer Res. 2022, 28, 677; 34716197 10.1158/1078-0432.CCR-21-1963

[62] a) C. Song , D. Liu , S. Liu , D. Li , I. Horecny , X. Zhang , P. Li , L. Chen , M. Miller , R. Chowdhury , M. Issa , R. Shen , Y. Yan , F. Zhang , L. Zhang , L. Zhang , C. Bai , J. Feng , L. Zhuang , R. Zhang , J. Li , H. Wilkinson , J. Liu , W. Tao , Sci. Rep. 2022, 12, 8579; 35595822 10.1038/s41598-022-12449-1 PMC9122897

[63] B. S. Pan , S. A. Perera , J. A. Piesvaux , J. P. Presland , G. K. Schroeder , J. N. Cumming , B. W. Trotter , M. D. Altman , A. V. Buevich , B. Cash , S. Cemerski , W. Chang , Y. Chen , P. J. Dandliker , G. Feng , A. Haidle , T. Henderson , J. Jewell , I. Kariv , I. Knemeyer , J. Kopinja , B. M. Lacey , J. Laskey , C. A. Lesburg , R. Liang , B. J. Long , M. Lu , Y. Ma , E. C. Minnihan , G. O'Donnell , et al., Science 2020, 369, aba6098. 10.1126/science.aba6098 32820094

[64] L. Wang , Z. Liang , Y. Guo , J. d. D. Habimana , Y. Ren , O. B. Amissah , O. Mukama , S. Peng , X. Ding , L. Lv , J. Li , M. Chen , Z. Liu , R. Huang , Y. Zhang , Y. Li , Z. Li , Y. Sun , Cell Death Dis. 2024, 15, 265. 38615022 10.1038/s41419-024-06638-1 PMC11016101

[65] E. N. Chin , C. Yu , V. F. Vartabedian , Y. Jia , M. Kumar , A. M. Gamo , W. Vernier , S. H. Ali , M. Kissai , D. C. Lazar , N. Nguyen , L. E. Pereira , B. Benish , A. K. Woods , S. B. Joseph , A. Chu , K. A. Johnson , P. N. Sander , F. Martinez‐Pena , E. N. Hampton , T. S. Young , D. W. Wolan , A. K. Chatterjee , P. G. Schultz , H. M. Petrassi , J. R. Teijaro , L. L. Lairson , Science 2020, 369, 993. 32820126 10.1126/science.abb4255

[66] H. Yang , W. S. Lee , S. J. Kong , C. G. Kim , J. H. Kim , S. K. Chang , S. Kim , G. Kim , H. J. Chon , C. Kim , J. Clin. Invest. 2019, 129, 4350. 31343989 10.1172/JCI125413 PMC6763266

[67] M. Chelvanambi , R. J. Fecek , J. L. Taylor , W. J. Storkus , J. ImmunoTherapy Cancer 2021, 9, 001906. 10.1136/jitc-2020-001906 PMC7852948 33526609

[68] J. Conlon , D. L. Burdette , S. Sharma , N. Bhat , M. Thompson , Z. Jiang , V. A. K. Rathinam , B. Monks , T. Jin , T. S. Xiao , S. N. Vogel , R. E. Vance , K. A. Fitzgerald , J. Immunol. 2013, 190, 5216. 23585680 10.4049/jimmunol.1300097 PMC3647383

[69] a) G. Schieven , J. Brown , J. Swanson , B. Stromko , C. Ho , R. Zhang , W. Li , H. Qiu , H. Sun , B. Fink , in Proceedings of the 33rd Annual Meeting & Pre‐Conference Programs of the Society for Immunotherapy of Cancer (SITC 2018) 2018, Washington, DC, USA p. 7;

[70] M. Adam , J. Yu , R. Plant , C. Shelton , H. Schmidt , J. Yang , Blood 2022, 140, 11829.

[71] W. Chang , M. D. Altman , C. A. Lesburg , S. A. Perera , J. A. Piesvaux , G. K. Schroeder , D. F. Wyss , S. Cemerski , Y. Chen , E. DiNunzio , A. M. Haidle , T. Ho , I. Kariv , I. Knemeyer , J. E. Kopinja , B. M. Lacey , J. Laskey , J. Lim , B. J. Long , Y. Ma , M. L. Maddess , B.‐S. Pan , J. P. Presland , E. Spooner , D. Steinhuebel , Q. Truong , Z. Zhang , J. Fu , G. H. Addona , A. B. Northrup , et al., J. Med. Chem. 2022, 65, 5675. 35332774 10.1021/acs.jmedchem.1c02197

[72] B. K. Allen , M. M. Kulkarni , B. Chamberlain , T. Dwight , C. Koh , R. Samant , F. Jernigan , J. Rice , D. Tan , S. Li , K. Marino , H. Huang , E. Chiswick , B. Tesar , S. Sparks , Z. Lin , T. D. McGee , I. Kolossváry , C. Lin , S. Shechter , H. Soutter , C. Bastos , M. Taimi , S. Lai , A. Petrin , T. Kane , S. Swann , H. Gardner , C. Winter , W. Sherman , (Preprint), bioRxiv 2022.05.23.493001, submitted: May 2022.

[73] K. J. Harrington , J. Brody , M. Ingham , J. Strauss , S. Cemerski , M. Wang , A. Tse , A. Khilnani , A. Marabelle , T. Golan , Ann. Oncol. 2018, 29, viii712.

[74] J. C. Moser , A. Alistar , E. Cohen , E. Garmey , S. Kazmi , T. Mooneyham , L. Sun , T. Yap , D. Mahalingam , J. ImmunoTherapy Cancer 2023, 11, A704. 10.1136/jitc-2025-011572 PMC12182170 40533265

[75] J. A. Marin‐Acevedo , E. O. Kimbrough , Y. Lou , J. Hematol. Oncol. 2021, 14, 45. 33741032 10.1186/s13045-021-01056-8 PMC7977302

[76] M. Yi , M. Niu , Y. Wu , H. Ge , D. Jiao , S. Zhu , J. Zhang , Y. Yan , P. Zhou , Q. Chu , K. Wu , J. Hematol. Oncol. 2022, 15, 142. 36209176 10.1186/s13045-022-01363-8 PMC9548169

[77] A. Ghaffari , N. Peterson , K. Khalaj , N. Vitkin , A. Robinson , J. A. Francis , M. Koti , Br. J. Cancer 2018, 119, 440. 30046165 10.1038/s41416-018-0188-5 PMC6133940

[78] L. Motedayen Aval , J. E. Pease , R. Sharma , D. J. Pinato , J. Clin. Med. 2020, 9, 3323. 33081170 10.3390/jcm9103323 PMC7602874

[79] F. Meric‐Bernstam , R. F. Sweis , S. Kasper , O. Hamid , S. Bhatia , R. Dummer , A. Stradella , G. V. Long , A. Spreafico , T. Shimizu , N. Steeghs , J. J. Luke , S. M. McWhirter , T. Müller , N. Nair , N. Lewis , X. Chen , A. Bean , L. Kattenhorn , M. Pelletier , S. Sandhu , Clin. Cancer Res. 2023, 29, 110. 36282874 10.1158/1078-0432.CCR-22-2235 PMC11188043

[80] J. J. Luke , S. A. Piha‐Paul , T. Medina , C. F. Verschraegen , M. Varterasian , A. M. Brennan , R. J. Riese , A. Sokolovska , J. Strauss , D. L. Hava , F. Janku , Clin. Cancer Res. 2023, 29, 2435. 37227176 10.1158/1078-0432.CCR-23-0118 PMC11225568

[81] a) K. Kawai , J. Miyazaki , A. Joraku , H. Nishiyama , H. Akaza , Cancer Sci. 2013, 104, 22; 23181987 10.1111/cas.12075 PMC7657210

[82] B. Temizoz , E. Kuroda , K. Ohata , N. Jounai , K. Ozasa , K. Kobiyama , T. Aoshi , K. J. Ishii , Eur. J. Immunol. 2015, 45, 1159. 25529558 10.1002/eji.201445132 PMC4671267

[83] S. Hajiabadi , S. Alidadi , Z. Montakhab Farahi , M. M. Ghahramani Seno , H. Farzin , A. Haghparast , Front. Immunol. 2023, 14, 1258691. 37901237 10.3389/fimmu.2023.1258691 PMC10611477

[84] A. M. Khalifa , T. Nakamura , Y. Sato , H. Harashima , Exp. Hematol. Oncol. 2024, 13, 36. 38553761 10.1186/s40164-024-00502-w PMC10981311

[85] S. Bhatnagar , V. Revuri , M. Shah , P. Larson , Z. Shao , D. Yu , S. Prabha , T. S. Griffith , D. Ferguson , J. Panyam , Cancers (Basel) 2022, 14, 6091. 36551577 10.3390/cancers14246091 PMC9777055

[86] E. Conde , E. Vercher , M. Soria‐Castellano , J. Suarez‐Olmos , U. Mancheño , E. Elizalde , M. L. Rodriguez , J. Glez‐Vaz , N. Casares , E. Rodríguez‐García , M. Hommel , G. González‐Aseguinolaza , I. Uranga‐Murillo , J. Pardo , G. Alkorta , I. Melero , J. Lasarte , S. Hervas‐Stubbs , Journal for ImmunoTherapy of Cancer 2021, 9, 003351. 10.1136/jitc-2021-003351 PMC8609946 34810235

[87] N. Xu , D. C. Palmer , A. C. Robeson , P. Shou , H. Bommiasamy , S. J. Laurie , C. Willis , G. Dotti , B. G. Vincent , N. P. Restifo , J. S. Serody , J. Exp. Med. 2020, 218, e20200844. 10.1084/jem.20200844 PMC7780733 33382402

[88] U. Uslu , L. Sun , S. Castelli , A. V. Finck , C.‐A. Assenmacher , R. M. Young , Z. J. Chen , C. H. June , Nat. Commun. 2024, 15, 3933. 38730243 10.1038/s41467-024-47692-9 PMC11087554

[89] Y. Da , Y. Liu , Y. Hu , W. Liu , J. Ma , N. Lu , C. Zhang , C. Zhang , OncoImmunology 2022, 11, 2054105. 35371622 10.1080/2162402X.2022.2054105 PMC8967397

[90] S. Kim , L. Li , Z. Maliga , Q. Yin , H. Wu , T. J. Mitchison , ACS Chem. Biol. 2013, 8, 1396. 23683494 10.1021/cb400264n PMC3815523

[91] J. Wu , N. Dobbs , K. Yang , N. Yan , Immunity 2020, 53, 115. 32640258 10.1016/j.immuni.2020.06.009 PMC7365768

[92] B. Larkin , V. Ilyukha , M. Sorokin , A. Buzdin , E. Vannier , A. Poltorak , J. Immunol. 2017, 199, 397. 28615418 10.4049/jimmunol.1601999 PMC5525333

[93] M. F. Gulen , U. Koch , S. M. Haag , F. Schuler , L. Apetoh , A. Villunger , F. Radtke , A. Ablasser , Nat. Commun. 2017, 8, 427. 28874664 10.1038/s41467-017-00573-w PMC5585373

[94] S. Li , B. Mirlekar , B. M. Johnson , W. J. Brickey , J. A. Wrobel , N. Yang , D. Song , S. Entwistle , X. Tan , M. Deng , Y. Cui , W. Li , B. G. Vincent , M. Gale , Y. Pylayeva‐Gupta , J. P. Y. Ting , Nature 2022, 610, 373. 36198789 10.1038/s41586-022-05254-3 PMC9875944

[95] N. Hashemi Goradel , M. Najafi , E. Salehi , B. Farhood , K. Mortezaee , J. Cell. Physiol. 2019, 234, 5683. 30341914 10.1002/jcp.27411

[96] a) H. Lemos , E. Mohamed , L. Huang , R. Ou , G. Pacholczyk , A. S. Arbab , D. Munn , A. L. Mellor , Cancer Res. 2016, 76, 2076; 26964621 10.1158/0008-5472.CAN-15-1456 PMC4873329

[97] R. Derynck , S. J. Turley , R. J. Akhurst , Nat. Rev. Clin. Oncol. 2021, 18, 9. 32710082 10.1038/s41571-020-0403-1 PMC9721352

[98] D. Zhang , D. Zhan , R. Zhang , Y. Sun , C. Duan , J. Yang , J. Wei , X. Li , Y. Lu , X. Lai , Sci. Rep. 2024, 14, 11593. 38773213 10.1038/s41598-024-62298-3 PMC11109281

[99] M. V. Guerin , F. Regnier , V. Feuillet , L. Vimeux , J. M. Weiss , G. Bismuth , G. Altan‐Bonnet , T. Guilbert , M. Thoreau , V. Finisguerra , E. Donnadieu , A. Trautmann , N. Bercovici , Nat. Commun. 2019, 10, 4131. 31511510 10.1038/s41467-019-11998-w PMC6739328

[100] S. E. Lee , G.‐Y. Jang , J. w. Lee , S. H. Park , H. D. Han , Y.‐M. Park , T. H. Kang , Cancer Immunol., Immunother. 2022, 71, 3029. 35610387 10.1007/s00262-022-03220-6 PMC10992159

[101] L. He , X. Xiao , X. Yang , Z. Zhang , L. Wu , Z. Liu , Cancer Lett. 2017, 402, 203. 28602976 10.1016/j.canlet.2017.05.026

[102] M. Motwani , S. Pesiridis , K. A. Fitzgerald , Nat. Rev. Genet. 2019, 20, 657. 31358977 10.1038/s41576-019-0151-1

[103] M. K. Ghosh , S. Roy , Mol. Biomed. 2024, 5, 4. 38253764 10.1186/s43556-023-00166-8 PMC10803705

[104] J. C. Kim , X. Liu , K. Fitzgerald , J. S. Eng , J. Orf , S. A. O'Brien , B. Belmontes , A. J. Casbon , S. V. Novitskiy , K. V. Tarbell , J. DeVoss , J. G. Egen , Cancer Immunol. Immunother. 2023, 72, 1327. 36394642 10.1007/s00262-022-03327-w PMC10110659

[105] A. Menz , J. Zerneke , F. Viehweger , S. Büyücek , D. Dum , R. Schlichter , A. Hinsch , A. A. Bawahab , C. Fraune , C. Bernreuther , M. Kluth , C. Hube‐Magg , K. Möller , F. Lutz , V. Reiswich , A. M. Luebke , P. Lebok , S. A. Weidemann , G. Sauter , M. Lennartz , F. Jacobsen , T. S. Clauditz , A. H. Marx , R. Simon , S. Steurer , E. Burandt , N. Gorbokon , S. Minner , T. Krech , Cancers 2024, 16, 2425. 39001487 10.3390/cancers16132425 PMC11240524

[106] a) X. Chen , Z. Xu , T. Li , A. Thakur , Y. Wen , K. Zhang , Y. Liu , Q. Liang , W. Liu , J.‐J. Qin , Y. Yan , Biomark. Res. 2024, 12, 2; 38185685 10.1186/s40364-023-00551-z PMC10773049

[107] T. T. Smith , H. F. Moffett , S. B. Stephan , C. F. Opel , A. G. Dumigan , X. Jiang , V. G. Pillarisetty , S. P. S. Pillai , K. D. Wittrup , M. T. Stephan , J. Clin. Invest. 2017, 127, 2176. 28436934 10.1172/JCI87624 PMC5451231

[108] B. B. Kocabas , K. Almacioglu , E. A. Bulut , G. Gucluler , G. Tincer , D. Bayik , M. Gursel , I. Gursel , J. Controlled Release 2020, 328, 587. 10.1016/j.jconrel.2020.09.040 32971199

[109] P. Zhang , A. Rashidi , J. Zhao , C. Silvers , H. Wang , B. Castro , A. Ellingwood , Y. Han , A. Lopez‐Rosas , M. Zannikou , C. Dmello , R. Levine , T. Xiao , A. Cordero , A. M. Sonabend , I. V. Balyasnikova , C. Lee‐Chang , J. Miska , M. S. Lesniak , Nat. Commun. 2023, 14, 1610. 36959214 10.1038/s41467-023-37328-9 PMC10036562

[110] T. Nakamura , T. Sato , R. Endo , S. Sasaki , N. Takahashi , Y. Sato , M. Hyodo , Y. Hayakawa , H. Harashima , Journal for ImmunoTherapy of Cancer 2021, 9, 002852. 10.1136/jitc-2021-002852 PMC8256839 34215690

[111] R. A. Bukhalid , J. R. Duvall , N. M. Cetinbas , K. C. Catcott , K. Avocetien , K. W. Bentley , S. Bradley , T. Carter , C.‐N. Chin , S. Clardy , Cancer Res. 2020, 80, 1158.

[112] Y.‐t. Wu , Y. Fang , Q. Wei , H. Shi , H. Tan , Y. Deng , Z. Zeng , J. Qiu , C. Chen , L. Sun , Z. J. Chen , Proc. Natl. Acad. Sci. USA 2022, 119, 2214278119. 10.1073/pnas.2214278119 PMC9894229 36442099

[113] J. R. Duvall , R. A. Bukhalid , N. M. Cetinbas , K. C. Catcott , K. Slocum , K. Avocetien , K. W. Bentley , S. Bradley , S. Clardy , S. D. Collins , E. Ditty , T. Eitas , B. D. Jones , E. W. Kelleher , W. Lee , T. Monnell , R. Mosher , M. Protopopova , L. Qin , P. Shaw , E. Ter‐Ovanesyan , J. D. Thomas , P. Wongthida , L. Xu , L. Yang , J. Zurita , D. Toader , M. Damelin , T. B. Lowinger , Cancer Res. 2021, 81, 1738.

[114] N. Malli Cetinbas , T. Monnell , J. Soomer‐James , P. Shaw , K. Lancaster , K. C. Catcott , M. Dolan , R. Mosher , C. Routhier , C.‐N. Chin , D. Toader , J. Duvall , R. Bukhalid , T. B. Lowinger , M. Damelin , Nat. Commun. 2024, 15, 5842. 38992037 10.1038/s41467-024-49932-4 PMC11239908

[115] C. Li , S. Mao , H. Zhao , M. He , M. Wang , Z. Liu , H. Wen , Z. Yu , B. Wen , D. Mahamadou , J. Tao , Y. Bu , W. Gao , D. Sun , (Preprint), bioRxiv 2024.02.14.580378, submitted: February 2024.

[116] J. Shi , C. Liu , S. Luo , T. Cao , B. Lin , M. Zhou , X. Zhang , S. Wang , T. Zheng , X. Li , Cell. Immunol. 2021, 366, 104384. 34182334 10.1016/j.cellimm.2021.104384

[117] S.‐S. Du , G.‐W. Chen , P. Yang , Y.‐X. Chen , Y. Hu , Q.‐Q. Zhao , Y. Zhang , R. Liu , D.‐X. Zheng , J. Zhou , J. Fan , Z.‐C. Zeng , Int. J. Radiat. Oncol. Biol., Phys. 2022, 112, 1243. 34986380 10.1016/j.ijrobp.2021.12.162

[118] W. Tang , W. Zhou , M. Ji , X. Yang , Cell Commun. Signaling 2024, 22, 202. 10.1186/s12964-024-01586-x PMC10986073 38566036

